# Shallow- and Deep-Water *Ophiura* Species Produce a Panel of Chlorin Compounds with Potent Photodynamic Anticancer Activities

**DOI:** 10.3390/antiox12020386

**Published:** 2023-02-05

**Authors:** Antonina Klimenko, Robin Huber, Laurence Marcourt, Dmitry Tabakaev, Alexey Koval, Salim Sh. Dautov, Tatyana N. Dautova, Jean-Luc Wolfender, Rob Thew, Yuri Khotimchenko, Emerson Ferreira Queiroz, Vladimir L. Katanaev

**Affiliations:** 1 Translational Research Center in Oncohaematology, Department of Cell Physiology and Metabolism, Faculty of Medicine, University of Geneva, Rue Michel-Servet 1, CH-1206 Geneva, Switzerland; 2 Institute of Life Sciences and Biomedicine, Far Eastern Federal University, 690090 Vladivostok, Russia; 3 School of Pharmaceutical Sciences, University of Geneva, Rue Michel-Servet 1, CH-1206 Geneva, Switzerland; 4 Institute of Pharmaceutical Sciences of Western Switzerland, University of Geneva, CMU, Rue Michel-Servet 1, CH-1206 Geneva, Switzerland; 5 Department of Applied Physics, Faculty of Sciences, University of Geneva, Rue de l’Ecole-De-Médecine 20, CH-1205 Geneva, Switzerland; 6 A.V. Zhirmunsky National Scientific Center of Marine Biology, Far East Branch of Russian Academy of Sciences, Palchevsky St. 17, 690041 Vladivostok, Russia

**Keywords:** marine organisms, chlorins, photodynamic therapy, brittle stars, Ophiuroidea, phototoxicity, cancer

## Abstract

A Pacific brittle star *Ophiura sarsii* has previously been shown to produce a chlorin (3*S*,4*S*)-14-Ethyl-9-(hydroxymethyl)-4,8,13,18-tetramethyl-20-oxo-3-phorbinepropanoic acid (ETPA) (**1**) with potent phototoxic activities, making it applicable to photodynamic therapy. Using extensive LC-MS metabolite profiling, molecular network analysis, and targeted isolation with de novo NMR structure elucidation, we herein identify five additional chlorin compounds from *O. sarsii* and its deep-sea relative *O. ooplax*: 10S-Hydroxypheophorbide a (**2**), Pheophorbide a (**3**), Pyropheophorbide a (**4**), (3*S*,4*S*,21*R*)-14-Ethyl-9-(hydroxymethyl)-21-(methoxycarbonyl)-4,8,13,18-tetramethyl-20-oxo-3-phorbinepropanoic acid (**5**), and (3*S*,4*S*,21*R*)-14-Ethyl-21-hydroxy-9-(hydroxymethyl)-4,8,13,18-tetramethyl-20-oxo-3-phorbinepropanoic acid (**6**). Chlorins **5** and **6** have not been previously reported in natural sources. Interestingly, low amounts of chlorins **1**–**4** and **6** could also be identified in a distant species, the basket star *Gorgonocephalus* cf. *eucnemis*, demonstrating that chlorins are produced by a wide spectrum of marine invertebrates of the class Ophiuroidea. Following the purification of these major *Ophiura* chlorin metabolites, we discovered the significant singlet oxygen quantum yield upon their photoinduction and the resulting phototoxicity against triple-negative breast cancer BT-20 cells. These studies identify an arsenal of brittle star chlorins as natural photosensitizers with potential photodynamic therapy applications.

## 1. Introduction

Photodynamic therapy (PDT) is a powerful alternative to other treatment options for cancer [1], dermatological aberrations [2], infectious diseases [3], or even cosmetics [4]. It is based on the activity of photosensitizers–compounds that are harmless unless excited with light of the proper wavelength. From the photoactivated state, a photosensitizer generates reactive oxygen species that are highly cytotoxic [5]. The systemic application of non-activated photosensitizers combined with the focal activation at the disease site with a laser makes PDT a unique therapeutic approach bordering between general and targeted treatments.

As with many other drugs, photosensitizers used in the clinic are derived from or inspired by nature [6]. Marine compounds are gaining interest as a source of new drugs [7,8,9]. Curiously, some natural metabolites with photosensitizer properties are used in their hosts for what can be considered a natural PDT. For example, bonellin, a chlorin-type compound synthesized by females of the sea worm *Bonellia viridis*, controls the sex determination of this polychete. Larvae of *B. viridis* develop into males when settled in the physical proximity of an adult female; the photodynamic action of bonellin is believed to play the key role in this process [10]. Photosensitizers also mediate the chemical defense function of their hosts against predators [10,11].

The number of factors determines whether a given photosensitizer can become a good candidate for medical PDT applications. One such factor is the excitation wavelength—the further in the red zone of the spectrum it is, the deeper the activating light can penetrate through biological tissues. Another is the singlet oxygen quantum yield of the compound; the higher, the better. Lack of toxicity at high concentrations of the non-activated photosensitizer is also an important factor. These and other pharmacokinetic/pharmacodynamic considerations frame the never-stopping search for and development of novel photosensitizers for PDT [12]. Much interest in this regard is devoted to chlorins that, in general, are powerful photosensitizers [13].

In our previous studies, we discovered the chlorin (3*S*,4*S*)-14-Ethyl-9-(hydroxymethyl)-4,8,13,18-tetramethyl-20-oxo-3-phorbinepropanoic acid (ETPA) from a Pacific brittle star *Ophiura sarsii* [14]. This major metabolite is easily purifiable and shows low cytotoxicity unless photoactivated. Red-light illumination escalates its potent submicromolar toxicity against a panel of cancer cell lines [15]. We further provided a proof-of-concept in vivo PDT application of ETPA in a mouse model of glioblastoma. These findings highlighted the promise of this brittle star chlorin for diverse PDT applications [14,15].

As ETPA is endogenously produced by *O. sarsii* [15], we wondered whether this brittle star might synthesize other chlorin compounds. As previously, the class Ophiuroidea has not been known to produce chlorins [11,16]. We also questioned whether other representatives of these marine invertebrates could constitute sources of natural photosensitizers for potential PDT applications. Here, we discover a family of chlorins in *O. sarsii* and other Ophiuroidea species, some of which were never identified as natural compounds previously. The strong phototoxic anticancer effects of these chlorins make them interesting candidates for further developments for PDT applications, as single compounds or as cocktails.

## 2. Materials and Methods

### 2.1. Materials

**Figure 1:**
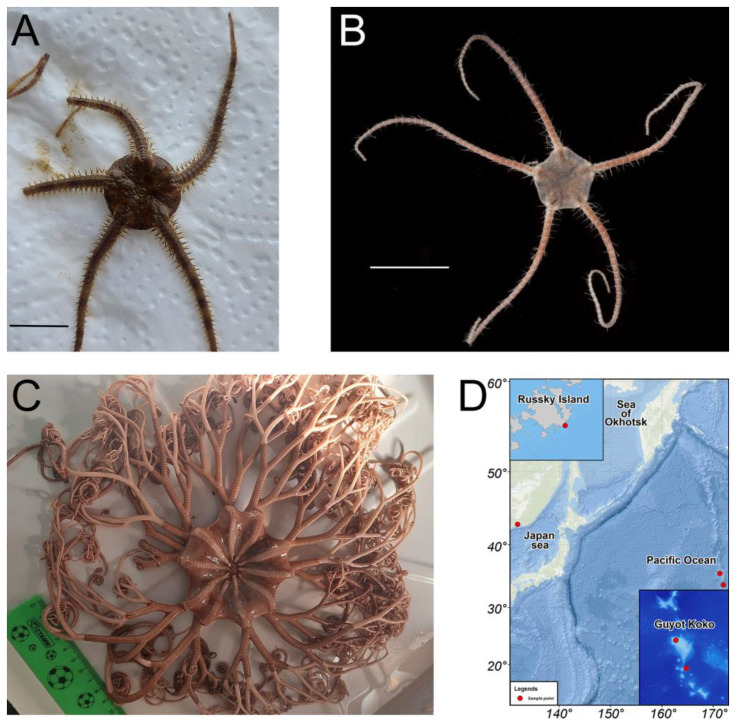
(**A**,**B**) Brittle stars *Ophiura sarsii* (**A**) and *Ophiura ooplax* (**B**). Scale bars: 1 cm. (**C**) Basket star *Gorgonocephalus* cf. *eucnemis*. (**D**) Map depicting the sample collection locations: Russky Island in the Japan sea for *O. sarsii*, Guyot Koko in the Pacific Ocean for *O. ooplax,* and *G. cf. eucnemis*.

Brittle stars *Ophiura sarsii* (Figure 1A) were collected at depths of 15–18 m in the Peter the Great Gulf (Sea of Japan) in May 2021; for location, see [15]. *Ophiura ooplax* [17] (Figure 1B) and the basket star *Gorgonocephalus* cf. *eucnemis* [18] (Figure 1C) were collected in July-August 2021 from the Koko guyot (Pacific Ocean, 35.60 N 171.23 E, depth 750 m, and 33.75 N, 171.92 E, depth 865 m, respectively, Figure 1D) using the remotely operated vehicle (ROV) Sub-Atlantic Comanche 18. These deep-sea samples were collected during the expedition of the A.V. Zhirmunsky National Scientific Center of Marine Biology (Far East Branch, Russian Academy of Sciences, Vladivostok, Russia) aboard the vessel R/V Akademik M. Lavrentyev (Cruise No. 94) to the seamounts of the Emperor Chain (Pacific Ocean) in July–September 2021. Sample collection was performed by the standards approved by the Ministry of Science and Higher Education (Russia); all efforts were made to minimize animal suffering. Species were identified by S.S.D. at the Laboratory of Embryology of the A.V. Zhirmunsky National Scientific Center of Marine Biology using morphological taxonomic characters. Freshly caught animals were washed twice under running water and frozen at −80 °C. One day after freezing, the animals were dried in a lyophilic dryer (Labconco FreeZone, Kansas City, MO, USA) and further stored at −23 °C.

### 2.2. Extraction

Before extraction, the material was lyophilized and homogenized using a Retsch MM301 mixer mill (Retsch, Eragny, France) at a vibration frequency of 1500 rpm for 2 min to obtain a homogeneous powder. 40 g of *O. sarsii* powder was poured into the Dionex ASE 350 solvent extraction system (Thermo Scientific, Bremen, Germany) and successively extracted by solvent of increasing polarity at 40 °C and using a holding time of 10 min for each cycle (50 mL/cycle). The solvents and their volumes were 800 mL of hexane, 250 mL of ethyl acetate (EtOAc), 250 mL of methanol (MeOH), and 200 mL of water. The extracts were dried using a rotary evaporator and/or lyophilization. The masses of the obtained extracts of *O. sarsii* were 459 mg of hexane, 51 mg of EtOAc, 1999 mg of MeOH, and 400 mg of water. The total yield of extraction was about 7%. The MeOH extract was further treated by liquid–liquid extraction to remove most of the polar compounds. The extract was solubilized in 120 mL of *n*-butanol and washed 3 times with 200 mL of H_2_O in a separating funnel. The butanol phase was dried on a rotary evaporator (250 mg).

A similar procedure was performed with *O. ooplax* and *G.* cf. *eucnemis*. A total of 29 g of *O. ooplax* were taken, ground into powder, and extracted in the ASE system (same parameters as described before) using 600 mL of hexane, 300 mL of EtOAc, 300 mL of MeOH, and 100 mL of water. The obtained extracts were dried using rotary evaporation and/or lyophilization. The weights of the obtained extracts of *O. ooplax* were 343 mg of hexane, 59.5 mg of EtOAc, 1598 mg of MeOH, and 267 mg of water. The total yield of extraction was about 8%. A total of 50 g of homogenized dry material of *G.* cf. *eucnemis* was extracted in the ASE system using solvents: 800 mL of hexane, 400 mL of EtOAc, 450 mL of MeOH, and 200 mL of water. The total weights of the extracts were hexane 1235 mg, EtOAc 116 mg, MeOH 2012 mg, and water 917 mg. The total yield of extraction was about 9%.

### 2.3. General Analytical Procedures

The optical rotations were measured in MeOH on a JASCO P-1030 polarimeter (Loveland, CO, USA) in a 10 cm tube. The UV–Vis and ECD spectra were recorded on a JASCO J-815 spectrometer (Loveland, CO, USA) in MeOH using a 1 cm cell. Scan speed was set at 200 nm/min in continuous mode between 800 and 190 nm with 3 accumulations. NMR spectroscopic data were recorded on a Bruker Avance Neo 600 MHz NMR spectrometer equipped with a QCI 5 mm cryoprobe and a SampleJet automated sample changer (Bruker BioSpin, Rheinstetten, Germany). One-dimensional and two-dimensional NMR experiments (^1^H, COSY, ROESY, and edited-HSQC) were recorded in DMSO-*d*_6_. Chemical shifts are reported in parts per million (δ) and coupling constants (*J*) in Hz. The residual DMSO-*d*_6_ signal (δ_H_ 2.50; δ_C_ 39.5) was used as internal standards for ^1^H and ^13^C NMR, respectively.

### 2.4. UHPLC-PDA-ELSD-MS Analysis

Chromatographic data were obtained on an ultra-high-performance liquid chromatography system equipped with a photodiode array, an evaporative light-scattering detector, and a single quadrupole detector using heated electrospray ionization (UHPLC-PDA-ELSD-MS) (Waters, Milford, MA, USA). The ESI parameters were the following: capillary voltage 800 V, cone voltage 15 V, source temperature 120 °C, and probe temperature 600 °C. Acquisition was performed in positive ionization mode with an *m/z* range of 150−1000 Da. The chromatographic separation was performed on an Acquity UPLC BEH C_18_ column (50 × 2.1 mm i.d., 1.7 μm; Waters) at 0.6 mL/min, 40 °C with H_2_O (A) and MeCN (B), both containing 0.1% formic acid as solvents. The gradient was carried out as follows: 5–100% B in 7 min, 1 min at 100% B, and a re-equilibration step at 5% B for 2 min. The ELSD temperature was fixed at 45 °C, with a gain of 9. The PDA data were acquired from 190 to 500 nm, with a resolution of 1.2 nm. The sampling rate was set at 20 points/s.

### 2.5. UHPLC-PDA-CAD-HRMS Analysis, Data-Processing, and Feature-Based Molecular Network Generation

Chromatographic data with high-resolution MS were obtained on a Waters Acquity UHPLC system equipped with a Q-Exactive Focus mass spectrometer (Thermo Scientific, Bremen, Germany), using heated electrospray ionization source (HESI-II). The chromatographic separation was carried out on an Acquity UPLC BEH C_18_ column (50 × 2.1 mm i.d., 1.7 μm; Waters) at 0.6 mL/min, 40 °C with H_2_O (A) and MeCN (B) both containing 0.1% formic acid as solvents. The gradient was carried out as follows: 5%–100% B in 7 min, 1 min at 100% B, and a re-equilibration step at 5% B in 2 min. The ionization parameters were the same as used in [19]. The raw UHPLC-HRMS/MS files were converted into mzXML files using the MSConvert software. The mzXML files were then processed using the open software MZmine (2.53) [20]. Mass detection step was performed using centroid mass detector with a noise level set at 5E4 for MS^1^ and 0 for MS^2^. The ADAP chromatogram builder was employed with a minimum group size of scans of 5, a minimum group intensity threshold of 1E4, a minimum highest intensity of 1E4, and an *m/z* tolerance of 8 ppm. The deconvolution was carried out with the ADAP (Wavelets) algorithm, using a signal-to-noise threshold of 10, a minimum feature height of 10, a coefficient/area threshold of 110, a peak duration range between 0.01 and 2.0 min, and a wavelet range between 0.01 and 0.10 min. The *m/z* and retention time (RT) ranges for MS^2^ scan pairing were, respectively, set to 0.005 Da and 0.1 min. The isotopes were grouped using the isotope peak grouper algorithm with an *m/z* tolerance of 8 ppm, an RT tolerance of 0.01 min, and a maximum charge of 2, using the most intense isotope as the representative one. The alignment was carried out with the join aligner with an *m/z* tolerance of 8 ppm, an RT tolerance of 0.1 min, and a weight tolerance for *m/z* and RT of 10 each. The MZmine aligned table was exported in MGF format for the processing of the Feature-based molecular network (FBMN). The spectral data were uploaded on the Global Natural Products Social (GNPS) platform [21]. A network was generated with a minimum cosine score of 0.65 and a minimum of 5 matching peaks. The experimental spectra were searched against GNPS’ spectral libraries. The obtained network was visualized in the software Cytoscape (3.7.2, Institute of Systems Biology, Seattle, WA, USA) [22].

### 2.6. Chromatographic Optimization and Semi-Preparative Isolation

The separation conditions of the MeOH and EtOAc extracts were optimized on an HP 1260 Agilent High-Performance liquid chromatography equipped with a photodiode array detector and an ELSD detector (HPLC-PDA-ELSD) (Agilent Technologies, Santa Clara, CA, USA). The chromatographic separation was performed on an XBridge C_18_ column (250 × 4.6 mm i.d., 5 μm; Waters) equipped with a C_18_ pre-column at 1 mL/min, with H_2_O (A) and MeCN (B), both containing 0.1% formic acid as solvents. The UV absorbance was measured at 280 and 360 nm, and UV–Vis spectra were recorded between 190 and 600 nm (step 2 nm). The optimized gradient used for the MeOH extract was from 45 to 100% of MeCN in 60 min, with 10 min of washing at 100% MeCN. The optimized gradient used for the EtOAc extract was from 55% to 90% MeCN in 60 min, with 10 min of washing at 100% MeCN. These chromatographic methods were geometrically transferred [23] to the semi-preparative scale on a Shimadzu system equipped with an LC-20 A module pumps, an SPD-20 A UV/VIS, a 7725I Rheodyne^®^ valve, and an FRC-40 fraction collector (Shimadzu, Kyoto, Japan). The separation was performed on an XBridge C_18_ column (250 mm × 19 mm i.d., 5 μm; Waters) equipped with a C_18_ pre-column cartridge holder (10 mm × 19 mm i.d., 5 μm; Waters) at 17 mL/min, with H_2_O (A) and MeCN (B) both containing 0.1% formic acid as solvents. The UV detection was set at 280 and 360 nm. The mixtures were injected into the semi-preparative HPLC column using a dry-load methodology developed in our laboratory [24]. Two injections of 50 mg were performed for the MeOH extract and one injection of 50 mg for the EtOAc extract. Six compounds were obtained (the yields for each compound are the sum of the compound isolated from the two extracts): **1** (2 mg, 70% purity), **2** (0.6 mg, 75% purity), **3** (3.8 mg, 85% purity), **4** (1.4 mg, 65% purity), **5** (0.3 mg, 75% purity), and **6** (0.8 mg, 70% purity). Purity estimation was based on the ^1^H-NMR spectra.

### 2.7. Description of the Isolated Compounds

(**1**): (3*S*,4*S*)-14-Ethyl-9-(hydroxymethyl)-4,8,13,18-tetramethyl-20-oxo-3-phorbinepropanoic acid (**ETPA**) **[14]** [α]^25^_D_ +300 (c 0.01, MeOH). UV (MeCN) λ_max_ (log ε) 269 (4.94), 320 (4.16), 375 (4.54) (sh), 393 (4.64) (sh), 408 (4.69), 504 (3.73), 535 (3.73), 604 (3.69), 661 (4.37) nm. ECD (c 20 μM, MeOH) λ_max_ ([θ]) 405 (+25,900), 660 (−22,200) nm. ^1^H NMR (DMSO-*d*_6_, 600 MHz) δ 0.33 (1H, s, NH15), 1.64 (3H, t, *J* = 7.6 Hz, H_3_-4b), 1.77 (3H, d, *J* = 7.2 Hz, H_3_-8a), 2.10 (1H, m, H-7a’’), 2.32 (1H, m, H-7b’’), 2.55 (1H, m, H-7b’), 2.61 (1H, m, H-7a’), 3.24 (3H, s, H_3_-3a), 3.38 (3H, overlapped, H_3_-1a), 3.62 (3H, s, H_3_-5a), 3.73 (2H, q, *J* = 7.6 Hz, H_2_-4a), 4.32 (1H, dt, *J* = 10.1, 3.0 Hz, H-7), 4.58 (1H, qd, *J* = 7.2, 3.0 Hz, H-8), 5.12 (1H, d, *J* = 19.4 Hz, H-10b’’), 5.22 (1H, d, *J* = 19.4 Hz, H-10b’), 5.74 (2H, s, H_2_-2a), 8.83 (1H, s, H-δ), 9.56 (1H, s, H-α), 9.73 (1H, s, H-β); ^13^C NMR (DMSO-*d*_6_, 151 MHz) δ 10.8 (CH_3_-1a), 11.0 (CH_3_-3a), 11.7 (CH_3_-5a), 17.5 (CH_3_-4b), 18.7 (CH_2_-4a), 22.9 (CH_3_-8a), 29.5 (CH_2_-7a), 30.9 (CH_2_-7b), 47.5 (CH_2_-10), 49.3 (CH-8), 51.1 (CH-7), 54.4 (CH_2_-2a), 93.6 (CH-δ), 97.0 (CH-α), 104.3 (CH-β), 106.0 (C-γ), 127.8 (C-5), 130.0 (C-6), 133.4 (C-1), 135.9 (C-3), 136.2 (C-12), 137.0 (C-15), 138.6 (C-2), 141.0 (C-11), 145.0 (C-4), 148.0 (C-16), 150.0 (C-14), 154.4 (C-13), 161.2 (C-17), 172.5 (C-18), 174.3 (C-7c), 195.3 (C-9). HR-ESI/MS analysis: *m/z* 539.2657 [M+H]^+^, (calcd for C_32_H_35_N_4_O_4_^+^, 539.2653, ∆ = 0.8 ppm). MS/MS spectrum: CCMSLIB00010128700. SMILES: OC(CC[C@H]1[C@@H](C2=N/C1=C(CC3=O)\C4=C3C(C)=C(N4)/C=C5N=C(C(C)=C\5CC)/C=C(N/6)/C(CO)=C(C6=C\2)C)C)=O. ^1^H, COSY, ^13^C-DEPTQ, HSQC, HMBC, and ROESY NMR spectra of compound **1** are shown in Supplementary Figures S1–S6, respectively.

(**2**): (3*S*,4*S*,21*S*)-9-Ethenyl-14-ethyl-21-hydroxy-21-(methoxycarbonyl)-4,8,13,18-tetramethyl-20-oxo-3-phorbinepropanoic acid (**(10*S*)-Hydroxypheophorbide a**) [25,26]: [α]^25^_D_ +65 (c 0.01, MeOH). UV (MeCN) λ_max_ (log ε) 275 (4.46), 329 (4.44) (sh), 370 (4.64) (sh), 408 (4.80), 500 (3.94), 535 (3.82), 609 (3.73), 665 (4.34) nm ECD (c 10 μM, MeOH) λ_max_ ([θ]) 406 (+20,400), 662 (−32,300) nm. ^1^H NMR (DMSO-*d*_6_, 600 MHz) δ 1.55 (3H, d, *J* = 7.4 Hz, H_3_-8a), 1.66 (3H, t, *J* = 7.7 Hz, H_3_-4b), 3.28 (3H, s, H_3_-3a), 3.47 (3H, s, H_3_-1a), 3.58 (3H, s, H_3_-10b), 3.71 (3H, s, H_3_-5a), 3.78 (2H, q, *J* = 7.7 Hz, H_2_-4a), 4.11 (1H, overlapped, H-7), 4.62 (1H, q, *J* = 7.4 Hz, H-8), 6.25 (1H, d, *J* = 11.7 Hz, H-2b’’), 6.44 (1H, d, *J* = 17.9 Hz, H-2b’), 7.74 (1H, s, OH10), 8.28 (1H, dd, *J* = 17.9, 11.7 Hz, H-2a), 9.01 (1H, s, H-δ), 9.60 (1H, s, H-α), 9.92 (1H, s, H-β); ^13^C NMR (DMSO-*d*_6_, 151 MHz) δ 11.0 (CH_3_-3a), 11.7 (CH_3_-5a), 12.0 (CH_3_-1a), 17.5 (CH_3_-4b), 18.7 (CH_2_-4a), 22.6 (CH_3_-8a), 49.3 (CH-8), 51.4 (CH-7), 52.6 (CH_3_-10b), 94.3 (CH-δ), 97.5 (CH-α), 104.9 (CH-β), 109.5 (C-γ), 123.5 (CH_2_-2b), 126.8 (C-6), 128.9 (CH-2a), 129.2 (C-5), 132.3 (C-1), 135.8 (C-2), 136.7 (C-3), 137.1 (C-15), 141.5 (C-11), 145.3 (C-4), 150.6 (C-14), 154.6 (C-13), 171.3 (C-10a), 173.3 (C-18), 189.2 (C-9). HR-ESI/MS analysis: *m/z* 609.2718 [M+H]^+^, (calcd for C_35_H_37_N_6_O_6_^+^, 609.2708, ∆ = 1.7 ppm). MS/MS spectrum: CCMSLIB00010128701. SMILES: OC(CC[C@H]1[C@@H](C2=N/C1=C([C@](C(OC)=O)(O)C3=O)\C4=C3C(C)=C(N4)/C=C5N=C(C(C)=C\5CC)/C=C(N/6)/C(C=C)=C(C6=C\2)C)C)=O. ^1^H, COSY, HSQC, HMBC, and ROESY NMR spectra of compound **2** are shown in Supplementary Figures S7–S11, respectively.

(**3**): (3*S*,4*S*,21*R*)-9-Ethenyl-14-ethyl-21-(methoxycarbonyl)-4,8,13,18-tetramethyl-20-oxo-3-phorbinepropanoic acid (**Pheophorbide a**) [27]: [α]^25^_D_ +400 (c 0.02, MeOH). UV (MeCN) λ_max_ (log ε) 275 (4.01), 328 (4.19) (sh), 374 (4.56) (sh), 406 (4.74), 502 (3.80), 536 (3.74), 605 (3.72), 665 (4.38) nm. ECD (c 15 μM, MeOH) λ_max_ ([θ]) 400 (+18,700), 662 (−10,700) nm. ^1^H NMR (DMSO-d_6_, 600 MHz) δ 0.41 (1H, s, NH15), 1.62 (3H, t, J = 7.7 Hz, H_3_-4b), 1.76 (3H, d, J = 7.3 Hz, H_3_-8a), 2.15 (1H, m, H-7a*’’*), 2.24 (1H, m, H-7b*’’*), 2.44 (1H, m, 7b*’*), 2.58 (1H, m, 7a*’*), 3.21 (3H, s, H_3_-3a), 3.43 (3H, s, H_3_-1a), 3.64 (3H, s, H_3_-5a), 3.70 (2H, q, J = 7.7 Hz, H_2_-4a), 3.83 (3H, s, H_3_-10b), 4.05 (1H, dt, J = 9.5, 2.8 Hz, H-7), 4.58 (1H, qd, J = 7.3, 2.2 Hz, H-8), 6.22 (1H, d, J = 11.6 Hz, H-2b*’’*), 6.40 (1H, d, J = 17.8 Hz, H-2b*’*), 6.42 (1H, s, H-10), 8.21 (1H, dd, J = 17.8, 11.6 Hz, H-2a), 8.91 (1H, s, H-*δ*), 9.44 (1H, s, H-*α*), 9.77 (1H, s, H-*β*), 12.05 (1H, s, COOH); ^13^C NMR (DMSO-d_6_, 151 MHz) δ 10.9 (CH_3_-3a), 11.7 (CH_3_-5a), 12.0 (CH_3_-1a), 17.4 (CH_3_-4b), 18.6 (CH_2_-4a), 22.9 (CH_3_-8a), 29.0 (CH_2_-7a), 30.7 (CH_2_-7b), 49.2 (CH-8), 50.8 (CH-7), 52.7 (CH_3_-10b), 64.2 (CH-10), 94.0 (CH-*δ*), 97.0 (CH-*α*), 105.0 (CH-*β*), 105.4 (C-*γ*), 123.3 (CH_2_-2b), 128.5 (C-6), 128.9 (C-5), 128.9 (CH-2a), 132.3 (C-1), 135.6 (C-2), 135.9 (C-12), 136.4 (C-3), 137.2 (C-15), 141.5 (C-11), 145.3 (C-4), 148.8 (C-16), 150.4 (C-14), 154.9 (C-13), 161.8 (C-17), 169.3 (C-10a), 173.2 (C-18), 174.0 (C-7c), 189.2 (C-9). Confirmed by comparison with the standard compound. HR-ESI/MS analysis: *m/z* 593.2745 [M+H]^+^, (calcd for C_35_H_37_N_4_O_5_^+^, 593.2758, ∆ = 2.3 ppm). MS/MS spectrum: CCMSLIB00010128702. SMILES: OC(CC[C@H]1[C@@H](C2=N/C1=C([C@@H](C(OC)=O)C3=O)\C4=C3C(C)=C(N4)/C=C5N=C(C(C)=C\5CC)/C=C(N/6)/C(C=C)=C(C6=C\2)C)C)=O. ^1^H, COSY, ^13^C-DEPTQ, HSQC, HMBC, and ROESY NMR spectra of compound **3** are shown in Supplementary Figures S12–S17, respectively.

(**4**): (3*S*,4*S*)-9-Ethenyl-14-ethyl-4,8,13,18-tetramethyl-20-oxo-3-phorbinepropanoic acid (**Pyropheophorbide a**) **[28]***:* [α]^25^_D_ +250 (c 0.01, MeOH). UV (MeCN) λ_max_ (log ε) 275 (4.17), 323 (4.22), 374 (4.48) (sh), 396 (4.62) (sh), 412 (4.68), 507 (3.73), 539 (3.70), 606 (3.64), 667 (4.20) nm. ECD (c 12 μM, MeOH) λ_max_ ([θ]) 412 (+12,600), 668 (−15,000) nm. ^1^H NMR (DMSO-d_6_, 600 MHz) δ 0.29 (1H, s, NH15), 1.64 (3H, t, J = 7.7 Hz, H_3_-4b), 1.78 (3H, d, J = 7.4 Hz, H_3_-8a), 2.12 (1H, m, H-7a*’’*), 2.33 (1H, m, H-7b*’’*), 2.56 (1H, m, H-7b*’*), 2.63 (1H, m, H-7a*’*), 3.25 (3H, s, H_3_-3a), 3.45 (3H, s, H_3_-1a), 3.64 (3H, s, H_3_-5a), 3.73 (2H, q, J = 7.7 Hz, H_2_-4a), 4.33 (1H, d, J = 9.6 Hz, H-7), 4.60 (1H, q, J = 7.8 Hz, H-8), 5.14 (1H, d, J = 19.5 Hz, H-10*’’*), 5.24 (1H, d, J = 19.5 Hz, H-10*’*), 6.23 (1H, d, J = 11.6 Hz, H-2b*’’*), 6.41 (1H, d, J = 17.7 Hz, H-2b*’*), 8.25 (1H, dd, J = 17.7, 11.6 Hz, H-2a), 8.91 (1H, s, H-*δ*), 9.48 (1H, s, H-*α*), 9.77 (1H, s, H-*β*); ^13^C NMR (DMSO-d_6_, 151 MHz) δ 11.0 (CH_3_-3a), 11.7 (CH_3_-5a), 12.0 (CH_3_-1a), 17.4 (CH_3_-4b), 18.7 (CH_2_-4a), 22.9 (CH_3_-8a), 29.4 (CH_2_-7a), 30.8 (CH_2_-7b), 47.5 (CH_2_-10), 49.3 (CH-8), 51.0 (CH-7), 106.2 (C-11), 123.1 (CH_2_-2b), 128.1 (C-5), 129.1 (CH-2a), 130.1 (C-6), 131.9 (C-1), 135.1 (C-2), 136.2 (C-3), 137.3 (C-15), 140.7 (C-11), 145.0 (C-4), 148.0 (C-9), 150.1 (C-14), 154.2 (C-13), 172.4 (C-18), 174.2 (C-7c), 195.3 (C-9). HR-ESI/MS analysis: *m/z* 535.2698 [M+H]^+^, (calcd for C_33_H_35_N_4_O_3_^+^, 535.2704, ∆ = 1.1 ppm). MS/MS spectrum: CCMSLIB00010128703. SMILES: OC(CC[C@H]1[C@@H](C2=N/C1=C(CC3=O)\C4=C3C(C)=C(N4)/C=C5N=C(C(C)=C\5CC)/C=C(N/6)/C(C=C)=C(C6=C\2)C)C)=O. ^1^H, COSY, HSQC, HMBC, and ROESY NMR spectra of compound **4** are shown in Supplementary Figures S18–S22, respectively.

(**5**): (3*S*,4*S*,21*R*)-14-Ethyl-9-(hydroxymethyl)-21-(methoxycarbonyl)-4,8,13,18-tetramethyl-20-oxo-3-phorbinepropanoic acid (**2-Hydroxymethyl-2-devinyl pheophorbide-a**) [29]: [α]^25^_D_ +300 (c 0.01, MeOH). UV (MeCN) λ_max_ (log ε) 272 (4.02), 324 (4.19) (sh), 371 (4.54), 399 (4.73), 500 (3.79), 533 (3.68), 600 (3.64), 663 (4.35) nm. ECD (c 15 μM, MeOH) λ_max_ ([θ]) 399 (+32,500), 661 (−20,800) nm. ^1^H NMR (DMSO-d_6_, 600 MHz) δ 0.47 (1H, s, NH15), 1.64 (3H, t, J = 7.7 Hz, H_3_-4b), 1.74 (3H, d, J = 7.4 Hz, H_3_-8a), 2.11 (1H, m, H-7a*’’*), 2.17 (1H, m, H-7a*’*), 2.20 (1H, m, H-7b*’’*), 2.44 (1H, m, H-7b*’*), 3.25 (3H, s, H_3_-3a), 3.38 (3H, s, H_3_-1a), 3.65 (3H, s, H_3_-5a), 3.74 (2H, q, J = 7.7 Hz, H_2_-4a), 3.82 (3H, s, H_3_-10b), 4.03 (1H, m, H-7), 4.57 (1H, q, J = 7.4 Hz, H-8), 5.74 (2H, d, J = 5.8 Hz, H_2_-2a), 5.84 (1H, t, J = 5.8 Hz, OH2a), 6.41 (1H, s, H-10), 8.84 (1H, s, H-*δ*), 9.58 (1H, s, H-*α*), 9.79 (1H, s, H-*β*); ^13^C NMR (DMSO-d_6_, 151 MHz) δ 10.8 (CH_3_-1a), 11.0 (CH_3_-3a), 11.7 (CH_3_-5a), 17.5 (CH_3_-4b), 18.7 (CH_2_-4a), 22.9 (CH_3_-8a), 49.3 (CH-8), 50.7 (CH-7), 52.6 (CH_3_-10b), 64.2 (CH-10), 93.8 (CH-*δ*), 97.5 (CH-*α*), 104.8 (CH-*β*), 128.1 (C-6), 128.6 (C-5), 134.0 (C-1), 136.2 (C-3), 136.9 (C-12), 137.1 (C-15), 139.1 (C-2), 141.6 (C-11), 145.4 (C-4), 148.8 (C-16), 150.4 (C-14), 155.1 (C-13), 169.2 (C-10a), 173.5 (C-18), 174.2 (C-7c), 189.2 (C-9). HR-ESI/MS analysis: *m/z* 597.2703 [M+H]^+^, (calcd for C_34_H_37_N_4_O_6_^+^, 597.2708, ∆ = 0.8 ppm). MS/MS spectrum: CCMSLIB00010128704. SMILES: OC(CC[C@H]1[C@@H](C2=N/C1=C([C@@H](C(OC)=O)C3=O)\C4=C3C(C)=C(N4)/C=C5N=C(C(C)=C\5CC)/C=C(N/6)/C(CO)=C(C6=C\2)C)C)=O. ^1^H, COSY, HSQC, HMBC, and ROESY NMR spectra of compound **5** are shown in Supplementary Figures S23–S27, respectively.

(**6**): (3*S*,4*S*,21*R*)-14-Ethyl-21-hydroxy-9-(hydroxymethyl)-4,8,13,18-tetramethyl-20-oxo-3-phorbinepropanoic acid (**(10*R*)-hydroxy-ETPA**): [α]^25^_D_ +200 (c 0.01, MeOH). UV (MeOH) λ_max_ (log ε) 283 (4.03), 312 (4.05), 360 (4.38) (sh), 398 (4.75), 494 (3.71), 500 (3.68), 525 (3.49), 606 (3.47), 665 (4.21) nm. ECD (c 12 μM, MeOH) λ_max_ ([θ]) 400 (+8080), 667 (−20,800) nm. ^1^H NMR (DMSO-d_6_, 600 MHz) δ 1.69 (3H, t, J = 7.7 Hz, H_3_-4b), 1.95 (3H, d, J = 7.3 Hz, H_3_-8a), 2.17 (3H, m, H_2_-7a, H-7b*’’*), 2.97 (1H, m, H-7b*’*), 3.30 (3H, s, H_3_-3a), 3.45 (3H, s, H_3_-1a), 3.82 (2H, q, J = 7.7 Hz, H_2_-4a), 3.88 (3H, s, H_3_-5a), 4.39 (1H, qd, J = 7.3, 3.1 Hz, H-8), 4.61 (1H, s, H-10), 4.94 (1H, dt, J = 12.1, 3.1 Hz, H-7), 5.80 (2H, s, H_2_-2a), 8.99 (1H, s, H-*δ*), 9.79 (1H, s, H-*α*), 9.90 (1H, s, H-*β*); ^1^H NMR (DMSO-d_6_, 151 MHz) δ 10.8 (CH_3_-1a), 11.0 (CH_3_-3a), 12.0 (CH_3_-5a), 17.7 (CH_3_-4b), 18.9 (CH_2_-4a), 23.6 (CH_3_-8a), 29.3 (CH_2_-7a), 33.8 (CH_2_-7b), 51.4 (CH-8), 53.9 (CH-7), 54.6 (CH_2_-2a), 75.1 (CH-10), 93.4 (CH-*δ*), 99.2 (CH-*α*), 102.7 (CH-*β*), 112.8 (C-6), 130.6 (C-5), 132.8 (C-1), 135.5 (C-12), 136.2 (C-3), 137.3 (C-15), 138.5 (C-2), 145.0 (C-4), 154.2 (C-13), 171.7 (C-18), 174.6 (C-7c). HR-ESI/MS analysis: *m/z* 555.2600 [M+H]^+^, (calcd for C_32_H_35_N_4_O_5_^+^, 555.2602, ∆ = 0.3 ppm). MS/MS spectrum: CCMSLIB00010128699. SMILES: OC(CC[C@H]1[C@@H](C2=N/C1=C([C@@H](O)C3=O)\C4=C3C(C)=C(N4)/C=C5N=C(C(C)=C\5CC)/C=C(N/6)/C(CO)=C(C6=C\2)C)C)=O. ^1^H, COSY, HSQC, HMBC, and ROESY NMR spectra of compound **6** are shown in Supplementary Figures S28–S32, respectively.

### 2.8. UV–Vis Spectrophotometry and Fluorescence Spectra

UV–Vis spectra were recorded on a spectrophotometer Agilent Cary 60 UV–Vis (Santa-Clara, CA, USA) in MeOH in the wavelength range from 200 to 800 nm. Fluorescence spectra were obtained from 50 μL of 500 μM stocks of compounds in anhydrous MeOH in black 384-well plates in Tecan M200Pro reader. Excitation wavelength was 350 nm (9 nm bandpass), and emission range was from 400 to 850 nm (20 nm bandpass) with the 2 nm step.

### 2.9. Relative Singlet Oxygen Quantum Yield Measurements and Calculations

To measure the relative singlet oxygen quantum yield via its phosphorescence intensity, a 637.5 nm laser diode beam was focused through one of the ports of a cuvette holder (Thorlabs CVH100/M) to a 2-mm cuvette by an A-coated lens L1 with 5 cm focal distance (Thorlabs LBF254-050-A). The cuvette was filled with a sample and fixed in the cuvette holder. Another C-coated lens L2 with 2 cm focal distance (Thorlabs LA1074-C) was mounted in the orthogonal port of the cuvette holder to collect and collimate the singlet oxygen phosphorescence. A bandpass filter IFB with a central wavelength of 1277 nm and a bandwidth of 20 nm (Oceanoptics) was installed between this lens and the cuvette. Another C-coated lens with 3 mm focal distance (Thorlabs) focused the collected phosphorescence to an optical fiber connected to a free-running multimode single-photon detector (SPAD, modification of ID Quantique ID220).

The number of detected phosphorescence photons *R_d_*, caused by singlet oxygen relaxation is as follows:

(1) $$R_{d}={\mathrm{ClN}}_{A}{\sigma R}_{L}\eta_{d}\eta_{l}\eta_{q}$$

where C is a concentration of a derivative in MeOH [mol/L], l is a cuvette length, $N_{A}$ is the Avogadro number, σ is a single-photon absorption cross-section [cm^2^] at the wavelength of laser excitation, $R_{L}$ is a laser excitation rate, i.e., the number of photons per second incident to the sample, $\eta_{d}$ is a combined collection and detection efficiency of the setup, $\eta_{l}$ is the amount of losses that are unrelated to the absorption by the sample (reflection by the cuvette’s glass, scattering and absorption in MeOH, etc.), and $\eta_{q}$ is a quantum yield of singlet oxygen. Concentrations of compounds were 0.95–1 μM, except for compound **6** (0.36 μM).

We start by finding $\eta_{l}$. We put the pure MeOH as a sample and measure the incident laser power before and after the cuvette. The ratio of these two gives us $\theta_{l}=0.85.$ We calculate $\sigma =\ln\left(10\right)\frac{10^{3}}{N_{A}}$ ϵ, where ϵ is a molar extinction coefficient or absorbance [cm^−1^M^−1^]. The concentration is given, however $\eta_{d}$ and $\eta_{q}$ are unknown. To factor out the $\eta_{d}$, we use commercially available Pheophorbide a (Santa Cruz Biotechnology, Texas, USA) with a known $\eta_{q}^{ph}=0.69$ and $\sigma^{ph}=2.71\times 10^{-17}\;$cm^2^ [30,31] as a reference and plug it back to 1, writing the equation for a singlet oxygen quantum yield of singlet oxygen as

(2) $$\eta_{q}=\frac{R_{d}}{R_{d}^{Ph}}\frac{C^{Ph}}{C}\frac{\sigma^{Ph}}{\sigma }\frac{R_{L}^{Ph}}{R_{L}}\eta_{q}^{Ph}$$

We averaged over 10 s measuring $R_{d}$ when the signal was distinguishable from the noise; otherwise, we measured during 100 s. Power of the laser was averaged over the same time periods. The value of relative singlet oxygen quantum yield for Pheophorbide a was double-checked using rhodamine 6G as a reference, obtaining the value of 0.677. We next used commercial pheophorbide a as a reference to measure how much power is absorbed by a layer l of pheophorbide a of concentration $C^{ph}$ under the excitation $I_{0}^{ph}$

(3) $$I_{abs}^{Ph}=C^{Ph}\sigma^{Ph}I_{0}^{Ph}$$

(4) $$I_{abs}=C\sigma I_{0},$$

where $I_{abs}$, C, σ, $I_{0}$, are absorbed power, concentration, absorption cross-section, and excitation power for all other compounds. We measure $I_{abs}$ or $I_{abs}^{Ph}$, subtracting the power value measure behind the cuvette from the excitation power $I_{0}$. So we can write that

(5) $$\sigma =\frac{I_{abs}}{I_{abs}^{Ph}}\frac{I_{0}^{Ph}}{I_{0}}\frac{C^{Ph}}{C}\sigma^{Ph}$$

The resulting relative quantum yields of singlet oxygen for the new compounds are given as $\eta_{q}^{res}$ in the text.

### 2.10. Cell Culture

Triple-negative human breast cancer cells of the BT-20 cell line were cultivated in DMEM medium + GlutaMAX supplement, with FBS 10% (both Gibco, Gaithersburg MD, USA) without antibiotics at 37 °C and 5% CO_2_. For all experiments, the minimum cell viability considered for plating was 95%, using the trypan blue test.

For the MTT cell survival assay, BT-20 cells were seeded in DMEM medium supplemented with 10% FBS and 1% gentamicin in a 384-well flat-bottomed transparent plate (Greiner, Monroe, NC, USA). Cells were seeded at a density of 3000 cells/well in a final medium volume of 30 μL of medium and cultured overnight in a CO_2_ incubator at 37 °C. The day after removal of the medium, 50 μL DMEM was added with serial dilutions of chlorins starting at 50 μg/mL. Cells with the compounds were incubated for 72 h. Cells in DMEM without added compounds served as positive controls; DMEM wells without cells served as negative controls. The resulting concentration of DMSO in the wells was maintained at 0.5%. Each experiment was conducted in four replicates.

### 2.11. Phototoxicity Assay

The phototoxic analysis was made using an LED lamp with a maximum wavelength of 730–870 nm and a power of 50W. Fluence was measured as 25 J/cm^2^ using a Newport Optical Power Meter 842-PE, MKS Instruments, Norwood, MA, USA. For phototoxicity studies, BT-20 cells were seeded in 384-well plates in DMEM medium supplemented with 10% FBS at a density of 3000 cells/well in a final medium volume of 30 μL and cultured overnight in a CO_2_ incubator at 37 °C. The next day, the chlorins were dissolved in DMSO and added to the cells in DMEM medium in series dilutions starting at 25 μg/mL for each. The cells were incubated for 2 h with the chlorin compounds to allow the cellular uptake of the chlorins, after which DMEM medium was replaced with 20 μL of transparent DPBS, and the plate was irradiated with a red LED lamp for 30 min. Next, 80 μL of the culture medium was added to each well for further cultivation. Wells with no cells were used as positive controls; wells without cells were used as negative controls. The resulting concentration of DMSO in the wells was maintained at 0.5%. After 72 h, the wells were emptied, and 30 µL of MTT reagent dissolved in PBS at 0.5 mg/mL, and after 3.5 h, the formazan crystals formed were solubilized with 50 µL of DMSO. In 5 min, optical density of the solution was measured at the wavelength of 590 nm by the Infinite M Plex multifunctional plate reader (Tecan, Switzerland).

## 3. Results

### 3.1. Isolation of Multiple Chlorins from Ophiura sarsii

In our previous work, a chlorin compound, (3*S*,4*S*)-14-Ethyl-9-(hydroxymethyl)-4,8,13,18-tetramethyl-20-oxo-3-phorbinepropanoic acid (ETPA), was isolated from a Pacific brittle star *Ophiura sarsii* [14]. In a subsequent study, this compound was shown to be endogenous to the species and to possess powerful phototoxicity against a panel of cancer cell lines; these findings led to a proof-of-concept application of ETPA for photodynamic therapy in a mouse glioblastoma model [15]. While ETPA is a major metabolite in *O. sarsii* [14,15], natural products are often present as a variety of structural analogs in plants and marine organisms [32].

This motivated a new sample collection to search for natural analogs of ETPA in May 2021. The samples were homogenized and extracted with solvents of increasing polarity (hexane, EtOAc, MeOH, and water). The MeOH extract was further treated by liquid–liquid extraction (butanol–water) to remove most of the salts and polar primary metabolites. In the extraction process, the hexane and water extracts were not considered for analysis because they are known to contain mainly lipids and saccharides, respectively. The EtOAc and MeOH (butanolic fraction) extracts were profiled by untargeted UHPLC-PDA-CAD-HRMS/MS in the data-dependent mode to highlight the presence of chlorin analogs. The obtained data were processed on the MZmine software and uploaded to the Global Natural Products Social (GNPS) platform [21] to generate a Feature-Based Molecular Network (FBMN). An FBMN represents “features” (an MS^2^ spectrum associated with a retention time) as nodes. Two nodes are connected by an edge if their spectral similarity (based on a cosine score) is above a defined level. This visualization allows the organization of a large amount of MS^2^ data and can facilitate the search for analogs.

**Figure 2:**
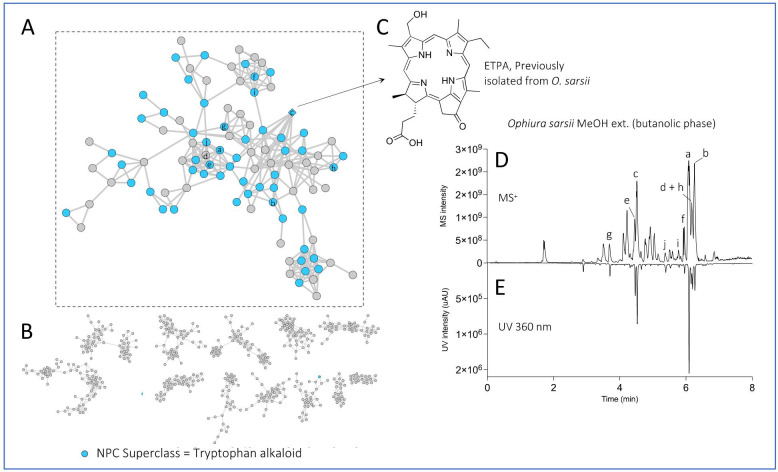
Feature-based Molecular Network of the EtOAc and MeOH (butanolic fraction) extracts of *Ophiura sarsii*. Nodes colored in blue belong to the “tryptophan alkaloid” predicted natural product superclass (NPClassifier). The previously isolated ETPA is displayed as a diamond. (**A**) The cluster containing chlorin ETPA and several related compounds. (**B**) Other major clusters of the Feature-Based Molecular Network. (**C**) Structure of the previously isolated ETPA. (**D**) UHPLC-MS profile of the MeOH extract (butanolic fraction) of *O. sarsii* with the 10 most intense ions of the chlorin cluster indicated with letters. (**E**) UHPLC-UV profile at 360 nm of the same extract. The same network mapped with the predicted natural product pathways is displayed in Supplementary Figure S33.

Such processing of the LC-MS/MS data on both extracts allowed the detection of 1202 features in positive ionization mode. The node ([M+H]^+^) associated with the previously isolated compound ETPA was highlighted (based on LC-MS analysis of the standard) in the FBMN by a diamond (Figure 2A,C). This compound is in a cluster of spectrally correlated features, which probably correspond to other chlorin analogs. Most of the features detected were found in both MeOH and EtOAc extracts. To further confirm the nature of the other nodes, the natural product classifier (NPClassifier) tool [33], which classifies compounds into a natural products ontology, was used on ETPA and assigns them to the superclass “tryptophan alkaloid”. The spectral data exported from MZmine were then processed with the SIRIUS software [34] and, in particular, the CANOPUS module, which allows chemical class prediction from an MS^2^ spectrum. The predicted chemical class of all features was then exported, and the “tryptophan alkaloid” superclass was mapped on the FBMN and confirmed that the nodes in the same cluster as the ETPA were mostly annotated with this superclass (Figure 2A). At a higher level, the predicted NP pathways (alkaloids, terpenoids, etc.) were also mapped on the FBMN (Supplementary Figure S33), which provides a quick overview of the chemical diversity of the extracts, which are mainly rich in fatty acids. This enabled us to highlight the 10 most intense features found in the chlorin cluster in the chromatogram of the MeOH extract (butanol fraction) of *O. sarsii* in view of the isolation (Figure 2D,E). These most intense MS peaks all have corresponding UV signals at 360 nm, typical of chlorins. This wavelength was, therefore, used to track the targeted peaks during the isolation process.

**Figure 3:**
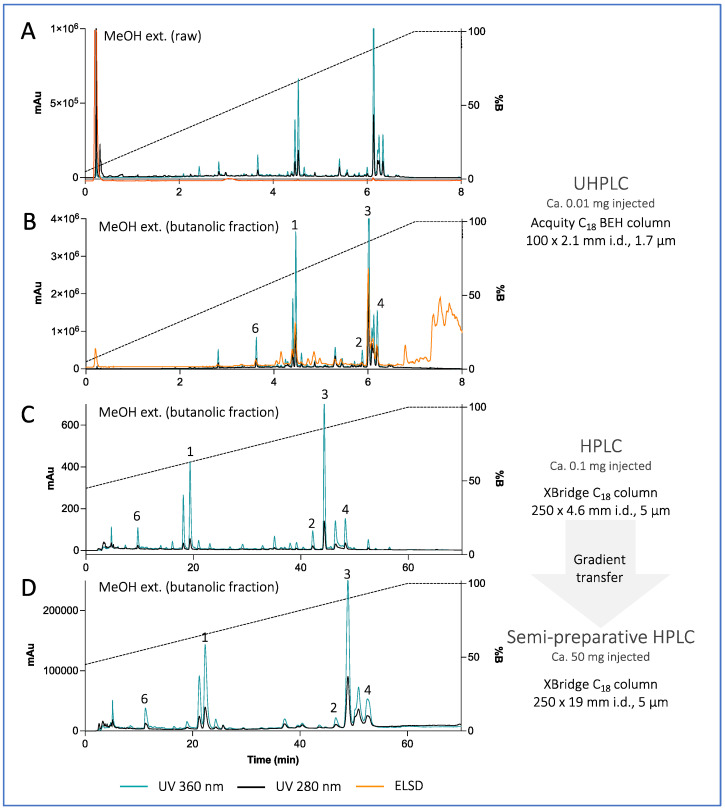
(**A**,**B**) UHPLC-PDA-ELSD analysis of the raw MeOH extract of *Ophiura sarsii* and its butanolic fraction. (**C**) Optimized HPLC-PDA-ELSD analysis of the MeOH extract (butanolic fraction) of *O. sarsii*. (**D**) semi-preparative HPLC-UV analysis after gradient transfer using a dry load injection. See Supplementary Figure S34 for the chromatograms of the EtOAc extract.

Targeted isolation of ETPA-related compounds was performed from both MeOH and EtOAc extracts. Chromatographic conditions were first optimized on an HPLC-PDA-ELSD instrument on a C_18_ column. The optimized gradients were geometrically transferred to the semipreparative HPLC scale [23] and injected in the column using a dry load method to maintain chromatographic resolution [24] (Figure 3 for the MeOH extract, Supplementary Figure S34 for the EtOAc extract). Six chlorins were isolated and fully characterized by HRMS, NMR, UV, ECD, and optical rotation using this approach. Among them, five were already known: (3*S*,4*S*)-14-Ethyl-9-(hydroxymethyl)-4,8,13,18-tetramethyl-20-oxo-3-phorbinepropanoic acid (ETPA) (**1**) that we previously isolated from this brittle star [14,15]; (10*S*)-Hydroxypheophorbide a (**2**) previously isolated from the microalgae *Chlorella regulans* [25] and the plant *Clerodendrum calamitosum* [26]; Pheophorbide a (**3**) previously isolated from the plant *Artemisia capillaris* [27]; Pyropheophorbide a (**4**) previously isolated from diverse species such as the plant *Atalantia monophyla* [35] *or the sea mussel Musculus senhousei* [28]; and (3*S*,4*S*,21*R*)-14-Ethyl-9-(hydroxymethyl)-21-(methoxycarbonyl)-4,8,13,18-tetramethyl-20-oxo-3-phorbinepropanoic acid (2-Hydroxymethyl-2-devinyl pheophorbide-a, (**5**) previously not reported from natural sources but cited in a synthesis patent [29]. Being less abundant and stable than the other chlorins, 2-Hydroxymethyl-2-devinyl pheophorbide-a was omitted from some of the following biophysical and cellular experiments.

Compound **6**, as compounds **1-5**, displayed the UV–Vis spectra typical for chlorin-type compounds (see below). The ^1^H NMR spectrum of compound **6** showed close similarities to that of compound **1**: three deshielded aromatic protons at δ_H_ 8.99 (1H, s, H-δ), 9.79 (1H, s, H-α), and 9.90 (1H, s, H-β), oxygenated methylene at δ_H_ 5.80 (2H, s, H_2_-2a), three deshielded methyl singlets at δ_H_ 3.30 (3H, s, H_3_-3a), 3.45 (3H, s, H_3_-1a), and 3.88 (3H, s, H_3_-5a), the typical ethylene group at δ_H_ 3.82 (2H, q, *J* = 7.7 Hz, H_2_-4a), and 1.69 (3H, t, *J* = 7.7 Hz, H_3_-4b), as well as a methyl doublet at δ_H_ 1.95 (3H, d, *J* = 7.3 Hz, H_3_-8a), and the two methine proton at δ_H_ 4.39 (1H, qd, *J* = 7.3, 3.1 Hz, H-8), and 4.94 (1H, dt, *J* = 12.1, 3.1 Hz, H-7). When compared to compound **1**, the two doublets from the methylene protons in C-10 (δ_H_ 5.12 and 5.22, *J* = 19.4 Hz) were missing and replaced by a singlet at δ_H_/δ_C_ 4.61/75.1 indicating a hydroxyl in C-10. The ROESY correlations from H_2_-7a to H-8 and H-10 indicate that the three methines, H-8, H-7, and H-10, were *trans*-oriented. The ROESY from H-7 to the methyl H_3_-8a and the deshielding of H-7 (δ_H_ 4.94) compared to Pheophorbide a (δ_H_ 4.05) confirmed the *trans* configuration of H-7 and H-8 and that the hydroxyl in C-10 was in the same side than H-7. The positive Cotton effect at 400 nm and negative at 667 nm confirmed that, like other chlorins isolated, the configuration at C-8 and C-7 of **1** was *S*, *S*. Compound **6** was thus identified as (3*S*,4*S*,21*R*)-14-Ethyl-21-hydroxy-9-(hydroxymethyl)-4,8,13,18-tetramethyl-20-oxo-3-phorbinepropanoic acid ((10*R*)-hydroxy-ETPA), that has never been reported. Furthermore, to our knowledge, this is the first report of a chlorin with hydrogen and a hydroxyl group in C-10 isolated from natural sources.

**Figure 4:**
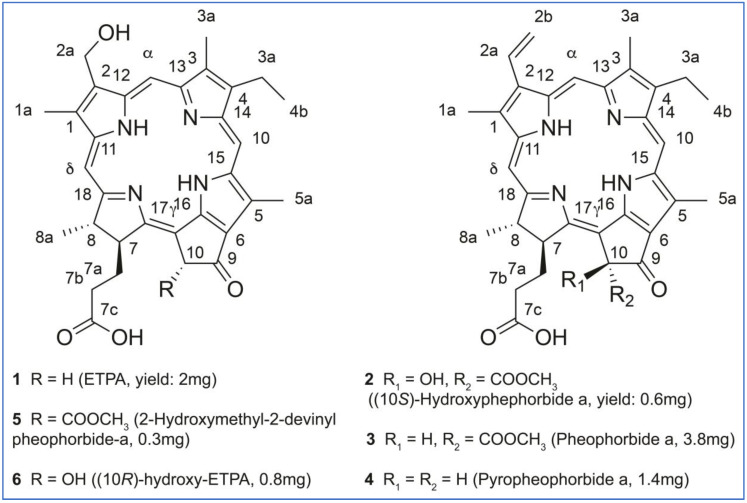
Structures and the yields of the chlorin compounds isolated from *O. sarsii*.

The elucidated structures of the chlorin compounds **1** to **6** from *O. sarsii* are shown in Figure 4. The Figure also provides the yields of these compounds, as isolated from EtOAc and/or MeOH extracts, starting from the dry mass of *O. sarsii* of 40 g. It can be seen that all six isolated chlorins are major metabolites, comparable in content to the ETPA we initially isolated from this brittle star.

### 3.2. Absorbance Spectra and Singlet Oxygen Production by Chlorins of O. sarsii

**Figure 5:**
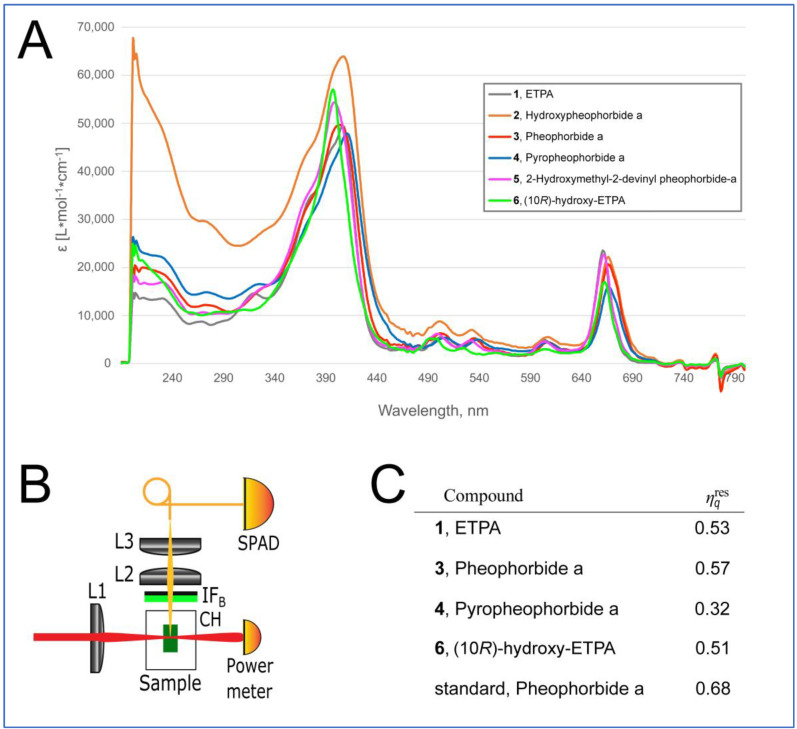
Physical properties of chlorin compounds from O. sarsii. (**A**) UV-Vis absorbance spectra of the chlorins. (**B**) Scheme of the device for measurement of the singlet oxygen generation. IF_B_*—*bandpass interference filter; L1*—*laser beam focusing lens; Sample*—*cell with chlorin solution; CH*—*cuvette holder; L2*—*singlet oxygen phosphorescence collecting lens; L3*—*phosphorescence focusing lens; SPAD*—*single-photon avalanche diode. (**C**) Quantification of the relative singlet oxygen quantum yield.

The isolated natural chlorin compounds show typical absorption and fluorescence spectra of chlorins such as the commercially available Pheophorbide a or the ETPA previously characterized by us [14,15] (Figure 5A and Supplementary Figures S35 and S36 for the UV–Vis, ECD, and fluorescence spectra). We next aimed at quantifying their relative singlet oxygen quantum yield using the device schematized in Figure 5B as described in Materials and Methods. Commercial Pheophorbide a was used as the standard.

The results of this analysis are depicted in Figure 5C. Surprisingly, the relative singlet oxygen quantum yield of Pheophorbide a isolated from *O. sarsii* was lower than that of the commercial analog (0.57 vs. 0.68 of commercial Pheophorbide a). Additionally, we previously used two different methods to calculate the singlet oxygen quantum yield of ETPA as 0.8 [15], which was considerably higher than the values we obtained for the current isolate (Figure 5C). We conclude that impurities in the current small-scale isolations of the chlorin compounds (see Materials and Methods) confound the correct quantification of the singlet oxygen quantum yield. However, the measured values still permit us to conclude that the arsenal of chlorins present in *O. sarsii* possesses significant singlet oxygen quantum yield properties.

### 3.3. Phototoxicity of O. sarsii Chlorins against Cancer Cells

**Figure 6:**
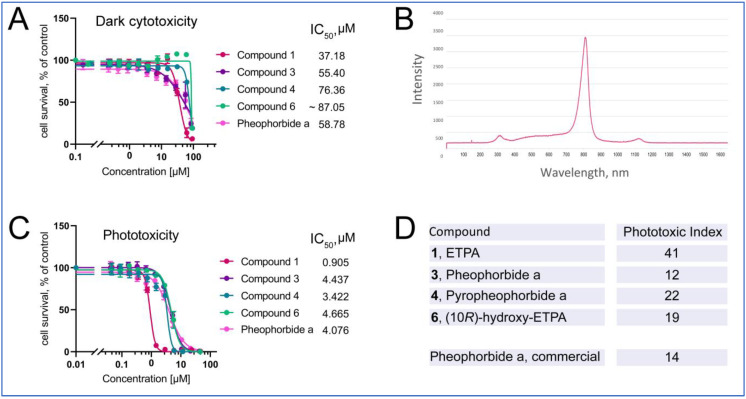
Phototoxicity of chlorins from *O. sarsii* against triple-negative breast cancer BT-20 cells. (**A**) Dark cytotoxicity of the compounds. (**B**) Emission spectrum of the LED lamp used in the phototoxicity experiment. (**C**) Phototoxicity of the compounds. (**D**) Phototoxic Index (PI) of the compounds. Commercial Pheophorbide a was used as a control in the experiments. The data are representatives of four independent experiments.

We next assessed the phototoxicity of the chlorin compounds isolated from *O. sarsii*, against the triple-negative breast cancer cell line BT-20. In the dark, when the chlorin compounds are not activated, their toxicities were low, with the IC_50_s in the range of 40–80 μM, corresponding to our previous findings on ETPA [14,15] (Figure 6A); commercial Pheophorbide a produced a similar value.

Photoactivation of the chlorins was achieved with an LED lamp, with the emission maximum partially covering the absorbance maximum of ca. 680 nm of chlorins (Figure 6B). Upon illumination with this lamp (see Materials and Methods for details), phototoxicity of the chlorin compounds was induced (Figure 6C) with the micomolar-to-submicromolar IC_50_s. Other isolates of compounds **1** and **3** give similar values (Supplementary Figure S37). The ratio of dark cytotoxicity IC_50_ to that of the phototoxicity gives the Phototoxic Index (PI)*—*the characteristic value of photosensitizers used in PDT. We find the PI values to vary from 12 to 41, being highest for ETPA among the tested compounds, in agreement with our previous findings [15].

### 3.4. Identification of Chlorins in a Deep-Sea Pacific Brittle Star O. ooplax and a Deep-Sea Pacific Basket Star Gorgonocephalus cf. eucnemis

Prior to our identification of the chlorin ETPA in *O. sarsii* [14,15], chlorin-type compounds were considered absent in the class Ophiurodea [11,16]. Although we have proven the endogenous (rather than exogenous, such as dietary) origin of ETPA in *O. sarsii* [15], the valid question is whether chlorins can be found in other representatives of the class, and if yes, whether the same set of chlorin compounds would be present in the other species. Additionally, an ecogeographical aspect could be considered: *O. sarsii* is mostly a shallow-waters brittle star that we collected in the Sea of Japan next to Russky Island (Figure 1D). Would deep-waters brittle stars living in other locations also carry chlorin compounds?

To address these questions, we took part in the expedition of the A.V. Zhirmunsky National Scientific Center of Marine Biology (Far East Branch, Russian Academy of Sciences) aboard the vessel R/V Akademik M. Lavrentyev (Cruise No. 94) to the seamounts of the Emperor Chain (Pacific Ocean) in July–September 2021. During this expedition, we collected invertebrate samples from depths of 750–1000 m near the Koko guyot (Pacific Ocean, Figure 1D). Among the samples, we collected and analyzed two representatives of the class Ophiuroidea: the brittle star *Ophiura ooplax* (Figure 1B) and the basket star *Gorgonocephalus* cf. *eucnemis* (Figure 1C).

*O. ooplax* (order Ophiurida, family Ophiuridae) has previously been identified at the coasts of Japan, Australia, and New Zealand, at depths ranging from 100 to 1170 m [17,36,37,38] and belongs to the same *Ophiura* genus as *O. sarsii*. In contrast, *Gorgonocephalus* cf. *eucnemis* [18] (order Phrynophiurida, family Gorgonocephalidae) is a distant member of the class Ophiurodea found in the northern hemisphere, including the Atlantic, Arctic, and Pacific Oceans at various depths reaching 4000 m (obis.org/taxon/124969) [39].

**Figure 7:**
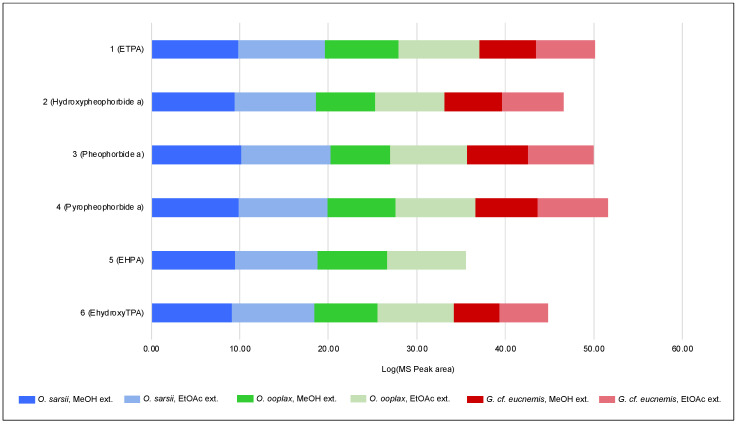
Log_10_ of the MS peak area (from UHPLC-HRMS/MS analysis) of the 6 isolated chlorins in the EtOAc and MeOH extracts of *Ophiura sarsii, Ophiura ooplax,* and *Gorgonocephalus* cf. *eucnemis*.

Samples of *O. ooplax* and *G.* cf. *eucnemis* were extracted in the same manner as described for *O. sarsii* (see Materials and Methods). EtOAc and MeOH extracts were profiled with UHPLC-PDA-CAD-HRMS/MS. The obtained data were again processed with MZmine with the EtOAc and MeOH extracts of *O. sarsii* (for the alignment step). The generated aligned feature table was exported, and the intensities (MS peak area) for each of the six isolated compounds in each extract were plotted (Figure 7). Most of the features corresponding to the six chlorin compounds were detected in all samples, but with important variations in MS intensities. In order to highlight the presence/absence of chlorins in the different samples, a logarithm was applied to the MS intensities for visualization purposes. The obtained results show that all isolated chlorins are found in both *O. sarsii* and *O. ooplax*. In the case of *G.* cf. *eucnemis*, five of the six chlorins are also detected (**1**, **2**, **3**, **4**, and **6**), but these are very minor compounds in the extracts. It should be noted, however, that the analysis we performed is only semi-quantitative, as the concentrations of the extracts were not standardized before the analysis. These data suggest that chlorins are not exclusive to the species *O. sarsii*, nor even to the genus *Ophiura*, but are likely present in different invertebrates of the class Ophiuroidea, albeit in different amounts and chemical representatives.

## 4. Discussion

Our findings characterize the marine invertebrates of the class Ophiuroidea as a rich source of natural photosensitizers. Six chlorins with photosensitizer properties emerge as major metabolites of the shallow- and deep-water brittle stars *Ophiura sarsii* and *Ophiura ooplax*, and five of the same chlorins can be found as minor metabolites in the basket star *Gorgonocephalus* cf. *eucnemis*. The class Ophiuroidea consists of >2000 known species (of which 400 are in the North Pacific), falling into 16 families [40]. A wider investigation involving more diverse brittle star and basket star (that represent the two orders within the class Ophiuroidea) species would be useful to draw conclusions on the chemodiversity and abundance of chlorins in these curious marine organisms. The role(s) they might play in their hosts is also open to further investigation.

We provide a detailed physical and biological characterization of the six Ophiuroidea chlorins, two of which have never been found in natural sources before. All chlorins emerge as photosensitizers with significant photodynamic properties against cancer cells, being well-tolerable without photoactivation by the red-spectrum light. These features make these natural chlorins interesting candidates for development in prospective PDT applications. The most powerful of the six, the chlorin ETPA, has been applied by us in a proof-of-concept in vivo PDT treatment of glioblastoma [15]—the most common brain tumor that is poorly treatable and has a bad prognosis, with a median survival below two years [41]. In the future, we plan to investigate this and the other Ophiuroidea chlorins in a panel of PDT applications in cancer, dermatology, and infectious disease indications [1,2,3]. Studying these natural chlorins, in solo or as cocktails/extracts, for cosmetics applications may also be attractive [4].

Such developments in natural chlorins will raise the need to solve the supply chain task—the common theme for marine-derived drugs [42]. Although *O. sarsii* represents an abundant shallow-waters brittle star in the North Pacific that is easily harvested, e.g., in the vicinity of the Russky Island, mariculture of this species appears as the best solution in case mass production becomes needed [43]. Chemical synthesis represents another possibility [44,45].

## 5. Conclusions

In conclusion, we discover an arsenal of photosensitizers in three species of North Pacific Ophiuroidea, shallow- and deep-water inhabitants. These natural chlorins possess attractive biophysical and anticancer properties. Our discoveries pave the way for further interdisciplinary investigations of the taxonomic chemodiversity of marine invertebrates and translational developments of their metabolites for diverse PDT applications.

## Data Availability

The MS^2^ spectrum of each isolated compound has its own accession number CCMSLIB00010128XXX on the Global Natural Product Social Molecular Networking (GNPS) (accessed via: https://gnps.ucsd.edu/ProteoSAFe/static/gnps-splash.jsp, accessed on 15 December 2022).

## Supplementary Materials

The following supporting information can be downloaded at: https://www.mdpi.com/article/10.3390/antiox12020386/s1, Figure S1: ^1^H NMR spectrum of compound **1**; Figure S2: COSY NMR spectrum of compound **1**; Figure S3: ^13^C-DEPTQ NMR spectrum of compound **1**; Figure S4: HSQC NMR spectrum of compound **1**; Figure S5: HMBC NMR spectrum of compound **1;** Figure S6: ROESY NMR spectrum of compound **1**; Figure S7: ^1^H NMR spectrum of compound **2**; Figure S8: COSY NMR spectrum of compound **2**; Figure S9: HSQC NMR spectrum of compound **2**; Figure S10: HMBC NMR spectrum of compound **2**; Figure S11: ROESY NMR spectrum of compound **2**; Figure S12: ^1^H NMR spectrum of compound **3**; Figure S13: COSY NMR spectrum of compound **3**; Figure S14: ^13^C-DEPTQ NMR spectrum of compound **3**; Figure S15: HSQC NMR spectrum of compound **3**; Figure S16: HMBC NMR spectrum of compound **3**; Figure S17: ROESY NMR spectrum of compound **3**; Figure S18: ^1^H NMR spectrum of compound **4**; Figure S19: COSY NMR spectrum of compound **4**; Figure S20: HSQC NMR spectrum of compound **4**; Figure S21: HMBC NMR spectrum of compound **4**; Figure S22: ROESY NMR spectrum of compound **4**; Figure S23: ^1^H NMR spectrum of compound **5**; Figure S24: COSY NMR spectrum of compound **5**; Figure S25: HSQC NMR spectrum of compound **5**; Figure S26: HMBC NMR spectrum of compound **5**; Figure S27: ROESY NMR spectrum of compound **5**; Figure S28: ^1^H NMR spectrum of compound **6**; Figure S29: COSY NMR spectrum of compound **6**; Figure S30: HSQC NMR spectrum of compound **6**; Figure S31: HMBC NMR spectrum of compound **6**; Figure S32: ROESY NMR spectrum of compound **6**; Figure S33: Feature-based Molecular Network of the EtOAc and MeOH (butanolic fraction) extracts of *Ophiura sarsii*; Figure S34: UHPLC-PDA-ELSD analysis of extracts of *Ophiura sarsii*; Figure S35: UV–Vis and ECD spectra of the isolated compounds; Figure S36: Fluorescence spectra of the isolated compounds; Figure S37: Phototoxicity of compounds **1** and **3** from MeOH and EtOAc extracts.
