# Supplementary material for: Shallow- and Deep-Water Ophiura Species Produce a Panel of Chlorin Compounds with Potent Photodynamic Anticancer Activities
Source: Antioxidants (Basel). 2023 Feb 5;12(2):386. doi: 10.3390/antiox12020386 (PMC9952619; doi:10.3390/antiox12020386)
Supplement: Supplementary file 1 [file antioxidants-12-00386-s001.zip › antioxidants-2134163-supplementary.pdf]

SUPPLEMENTARY MATERIALS include Supplementary Figures S1-S36.

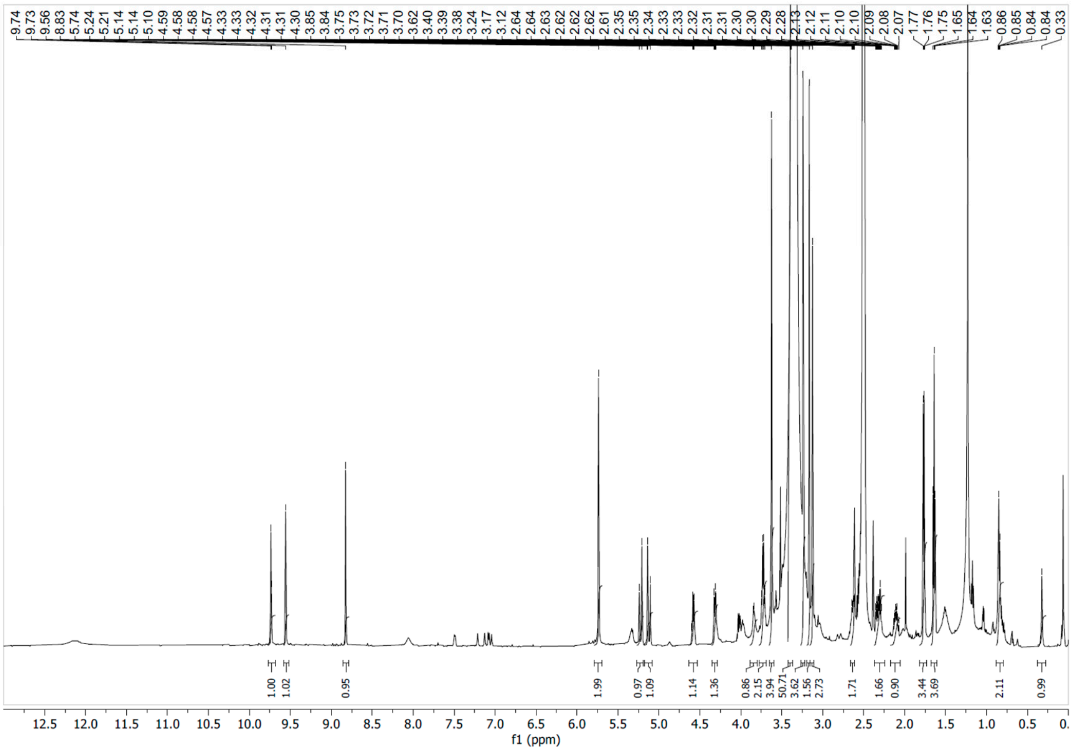

Supplementary Figure S1. ^{1}H NMR spectrum of compound **1** in DMSO-d_6 at 600 MHz.

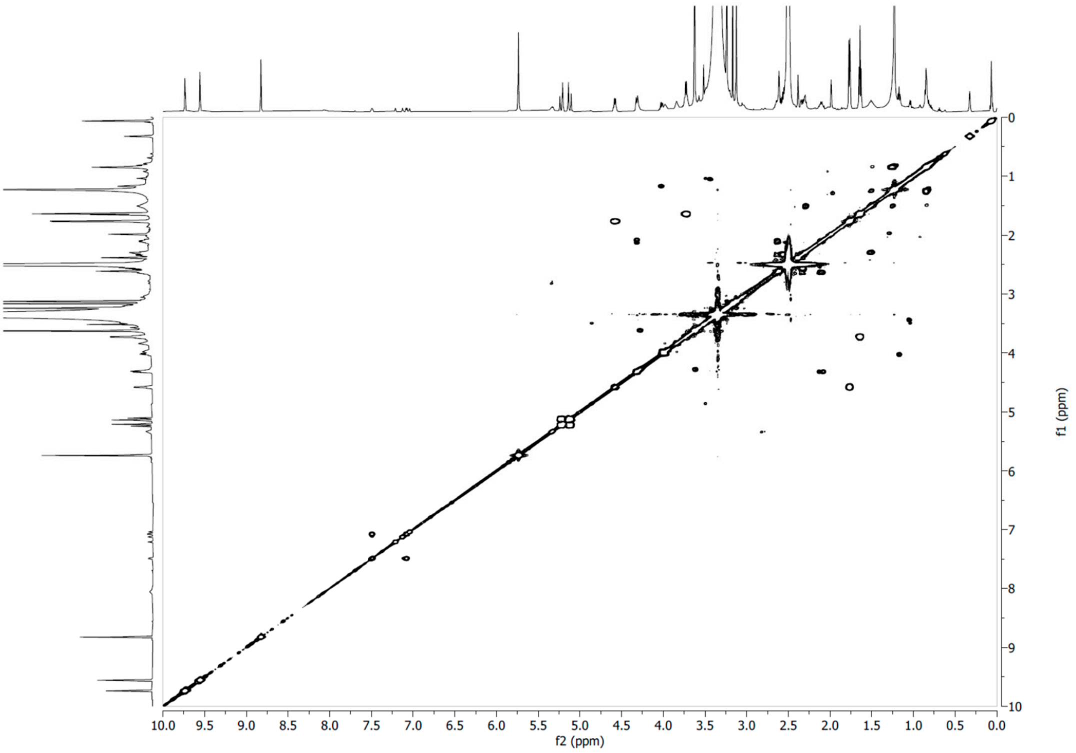

Supplementary Figure S2. COSY NMR spectrum of compound **1** in DMSO-d_6.

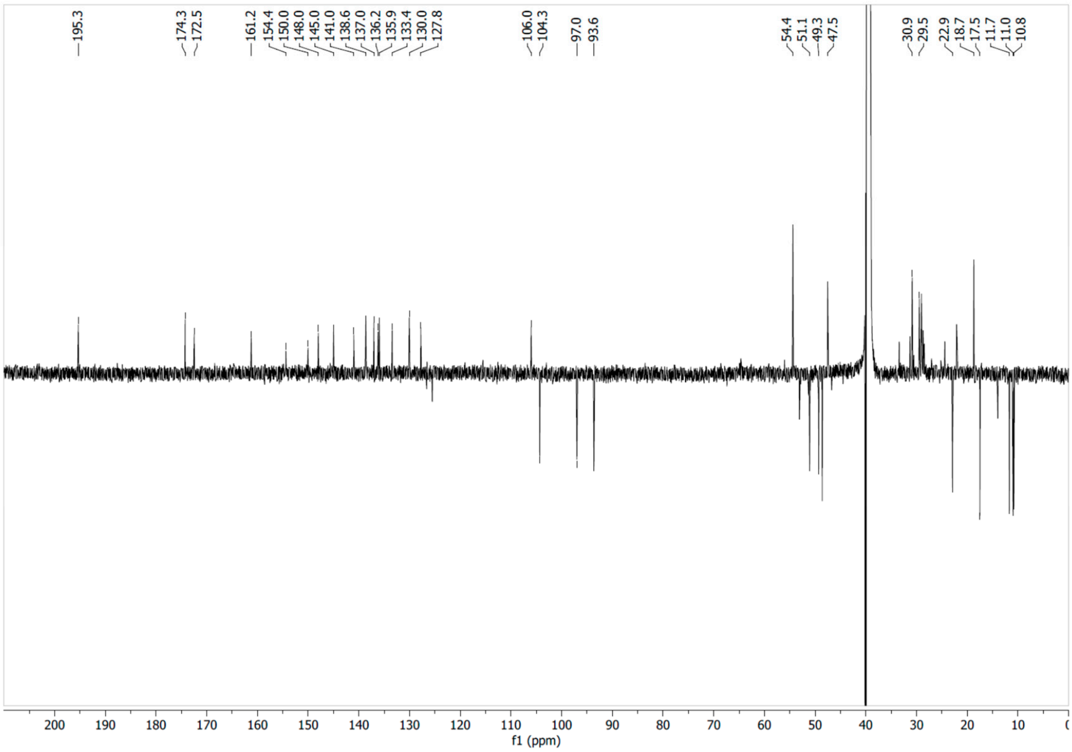

Supplementary Figure S3. ^{13}C-DEPTQ NMR spectrum of compound **1** in DMSO-d_6 at 151 MHz.

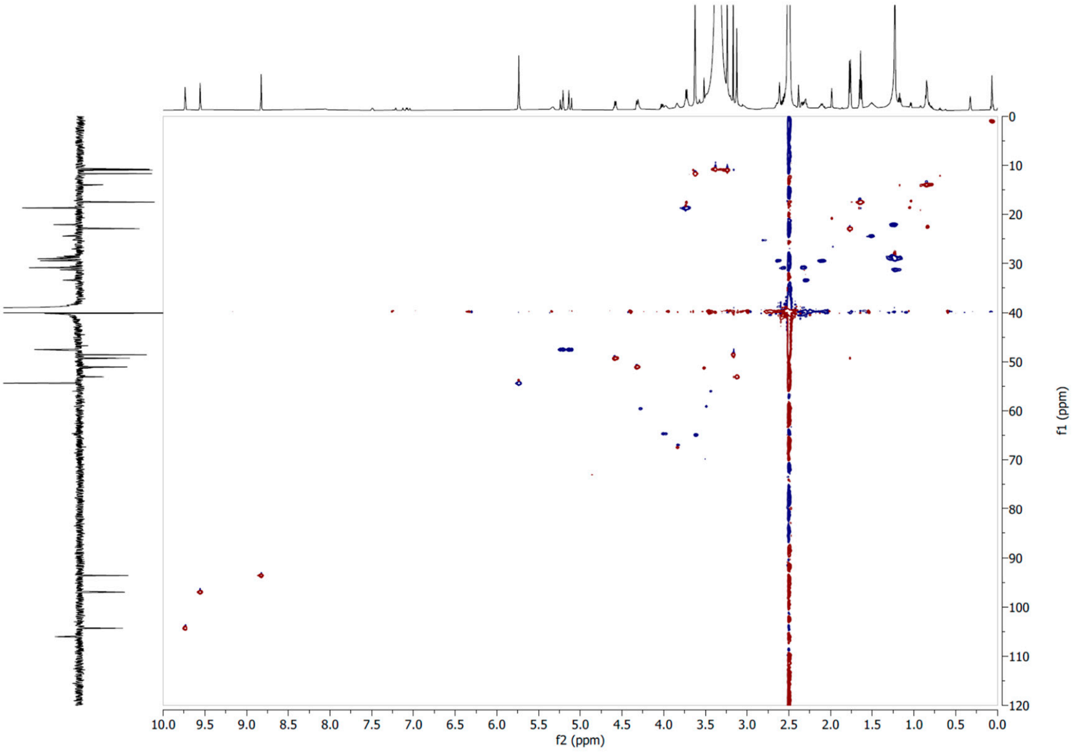

Supplementary Figure S4. Edited HSQC NMR spectrum of compound **1** in DMSO-d_6.

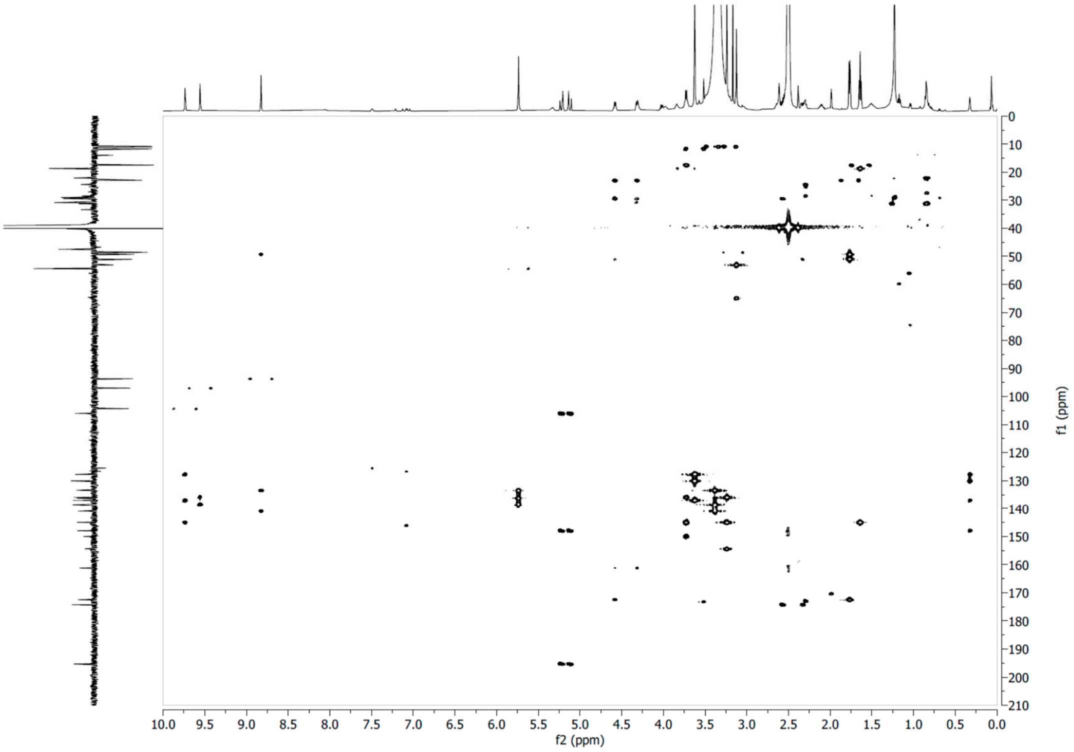

Supplementary Figure S5. HMBC NMR spectrum of compound **1** in DMSO-d_6.

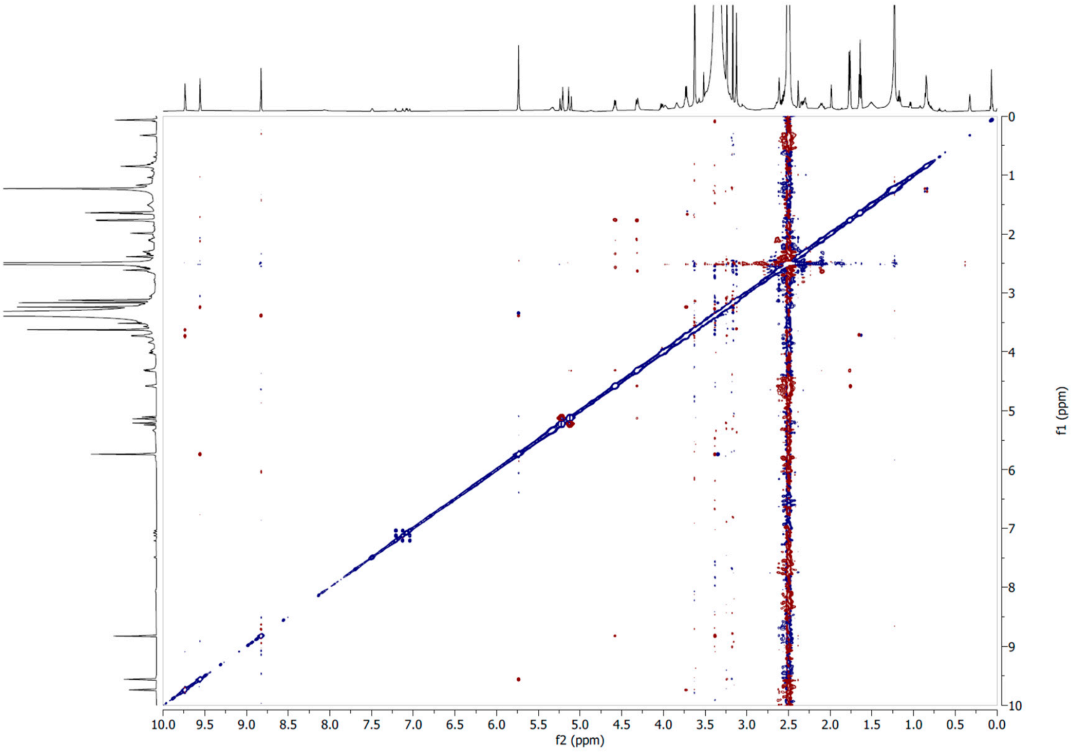

Supplementary Figure S6. ROESY NMR spectrum of compound **1** in DMSO-d_6.

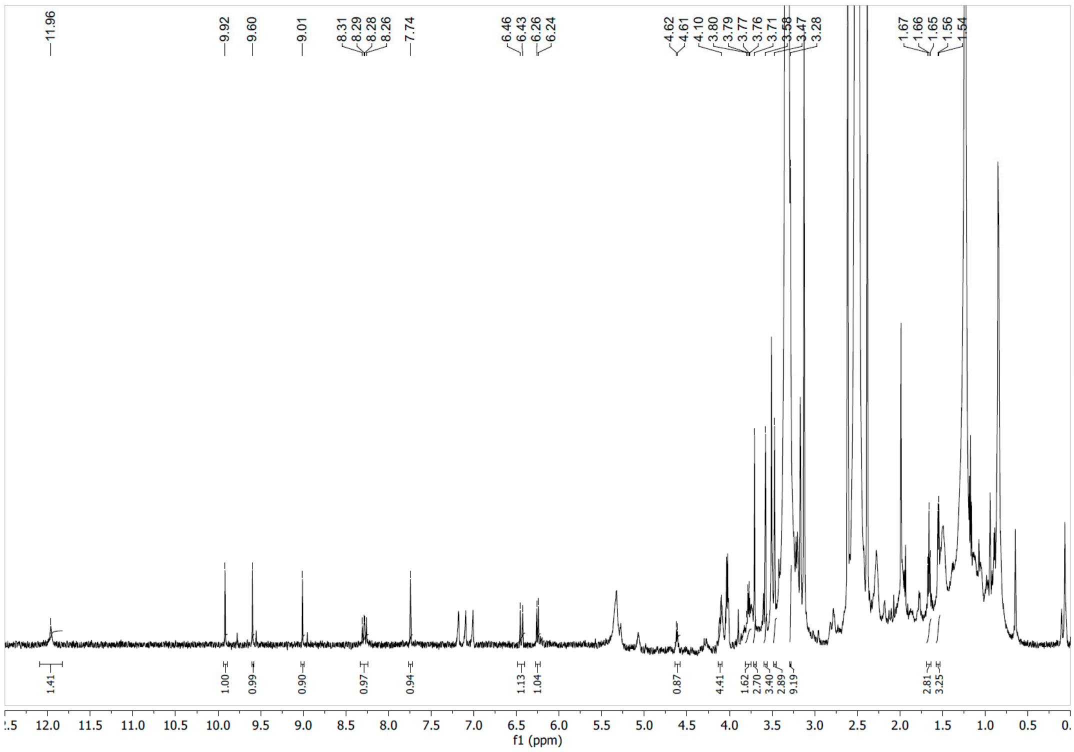

Supplementary Figure S7. ^{1}H NMR spectrum of compound **2** in DMSO-d_6 at 600 MHz.

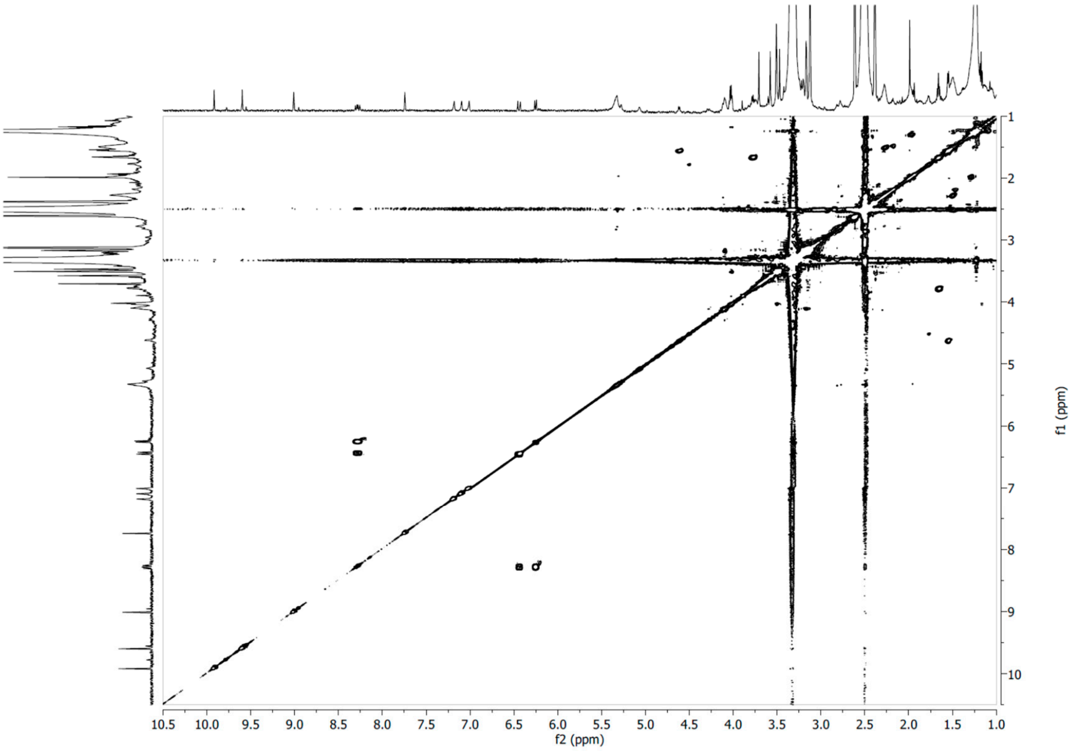

Supplementary Figure S8. COSY NMR spectrum of compound **2** in DMSO-d_6.

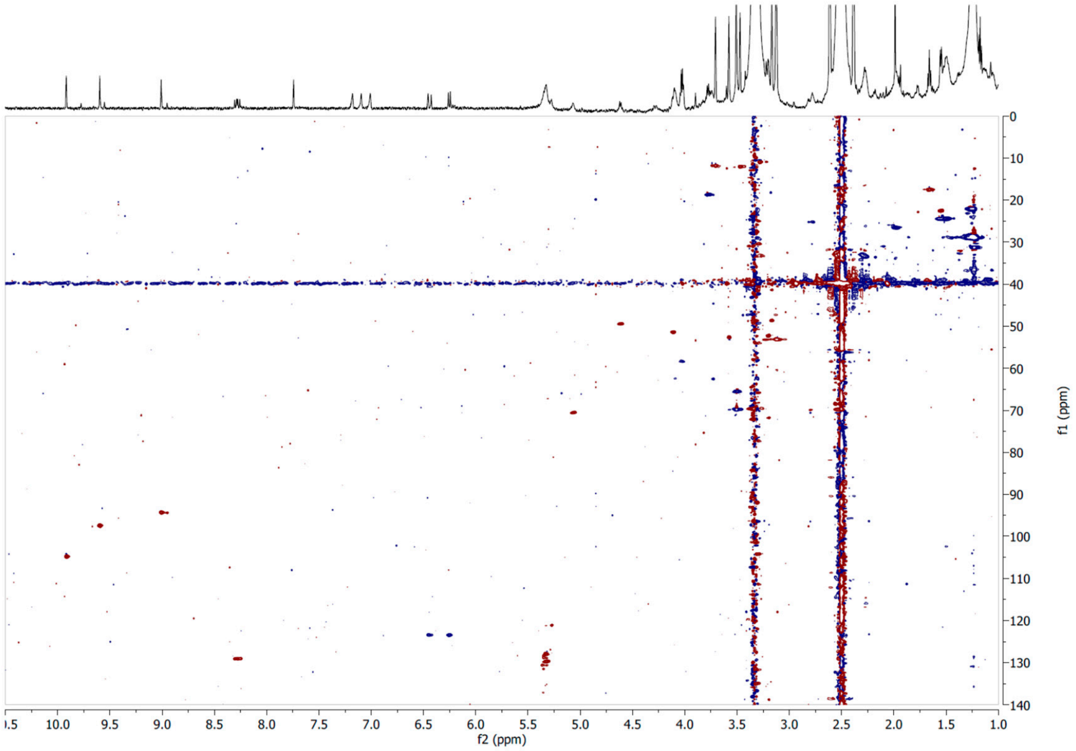

Supplementary Figure S9. Edited HSQC NMR spectrum of compound **2** in DMSO-d_6.

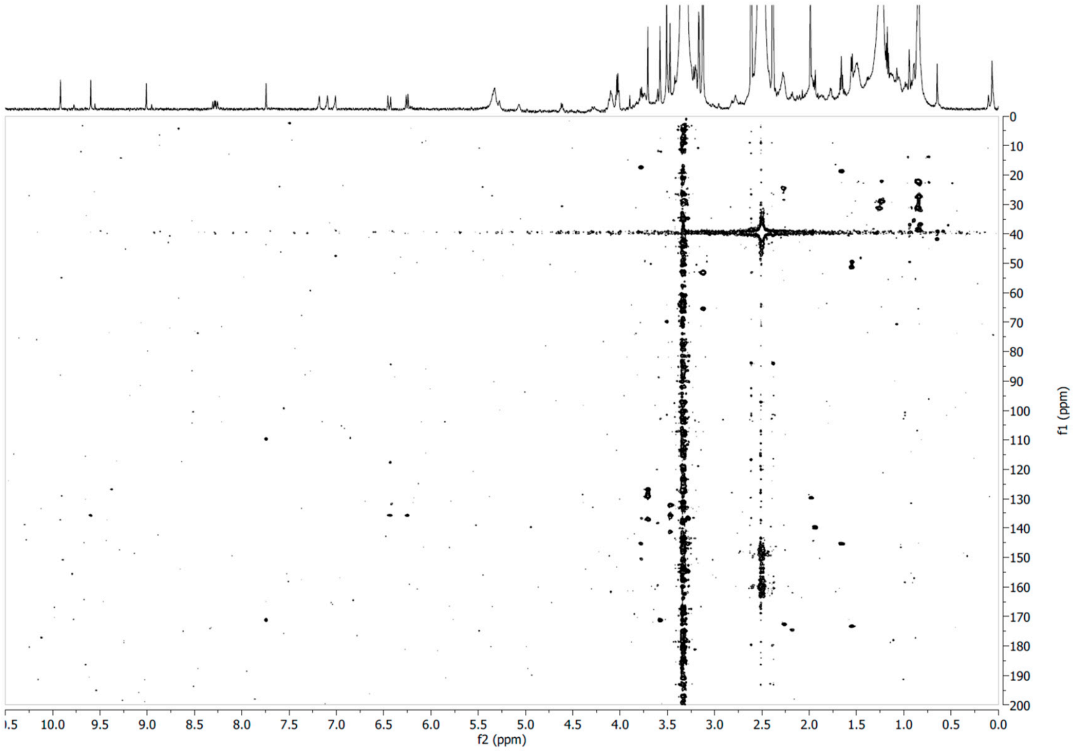

Supplementary Figure S10. HMBC NMR spectrum of compound **2** in DMSO-d_6.

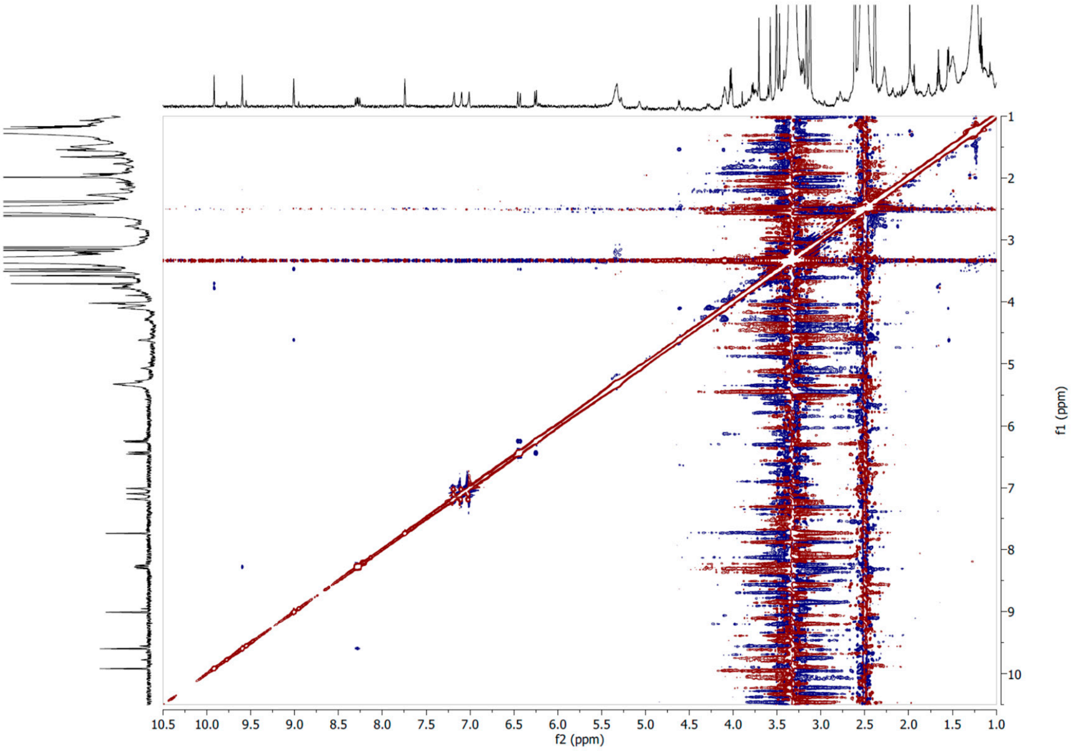

Supplementary Figure S11. ROESY NMR spectrum of compound **2** in DMSO-d_6.

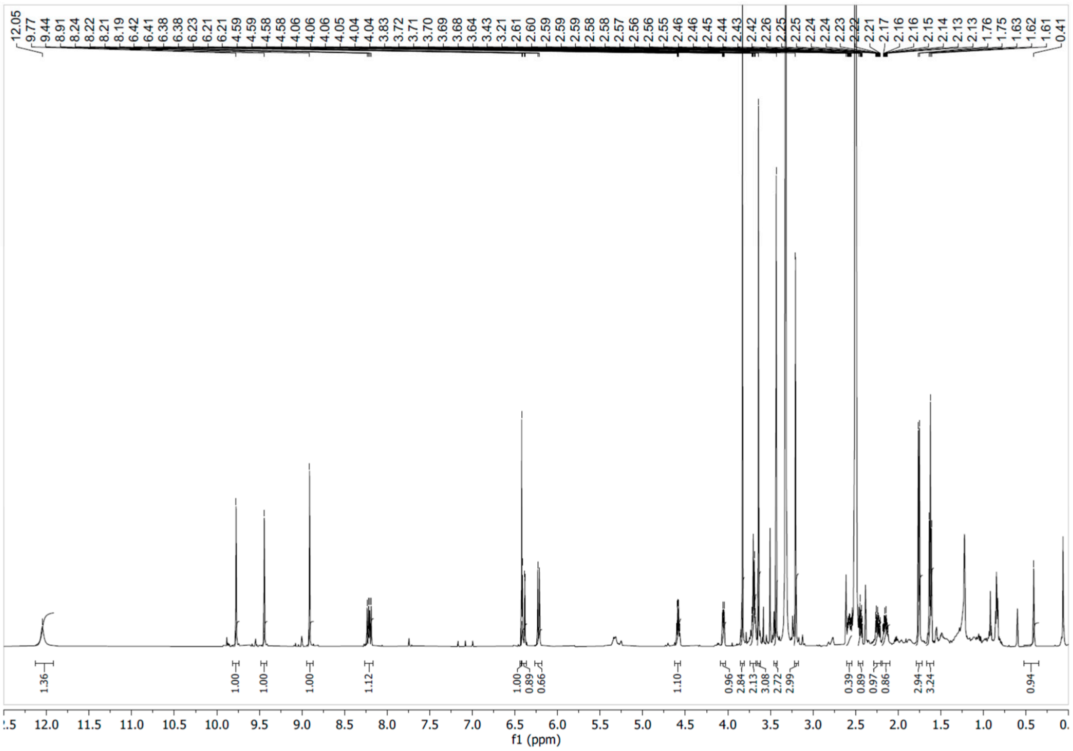

Supplementary Figure S12. ^{1}H NMR spectrum of compound **3** in DMSO-d_6 at 600 MHz.

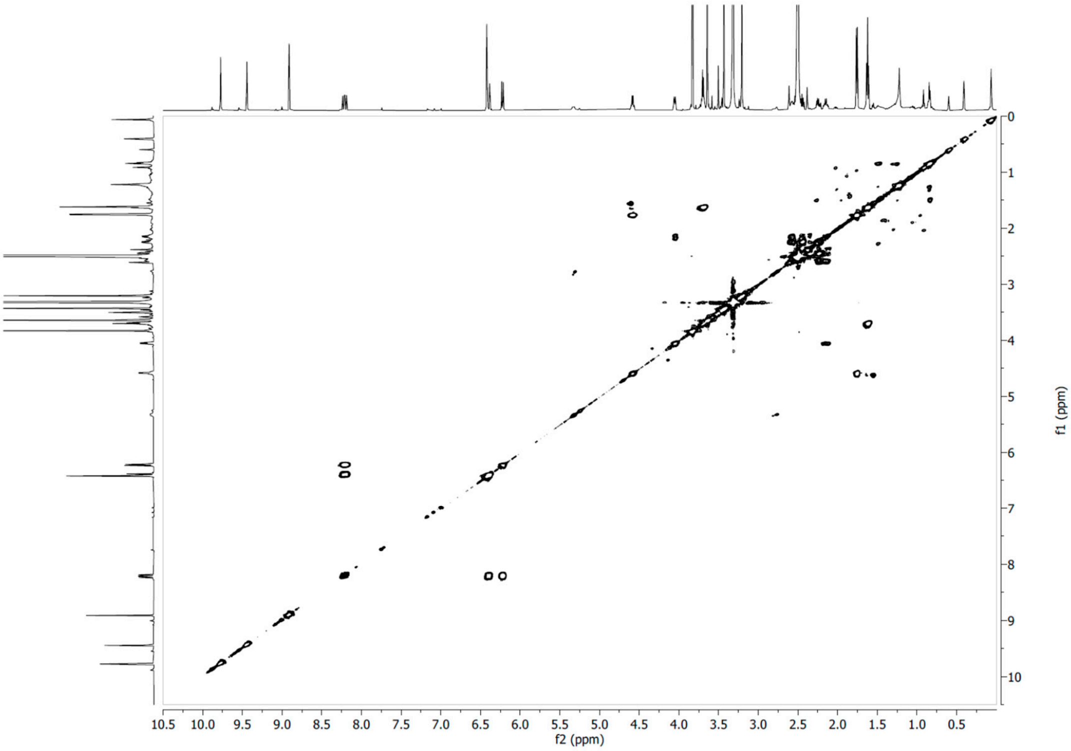

Supplementary Figure S13. COSY NMR spectrum of compound **3** in DMSO-d_6.

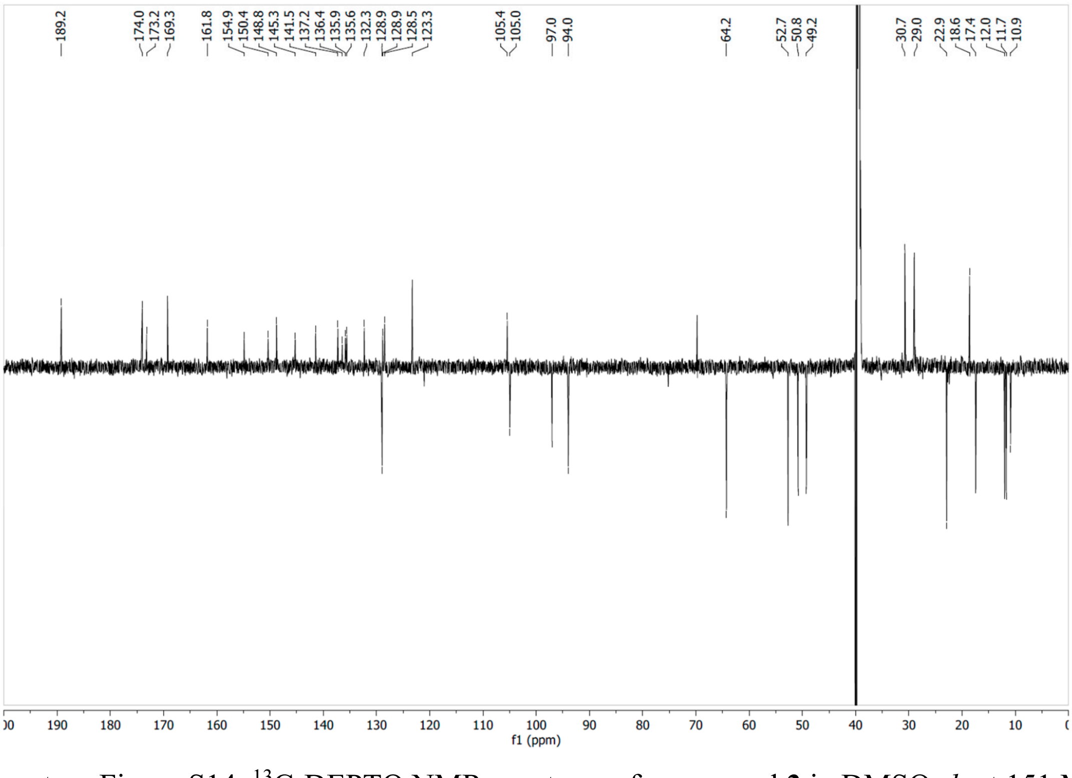

Supplementary Figure S14. ^{13}C-DEPTQ NMR spectrum of compound **3** in DMSO-d_6 at 151 MHz.

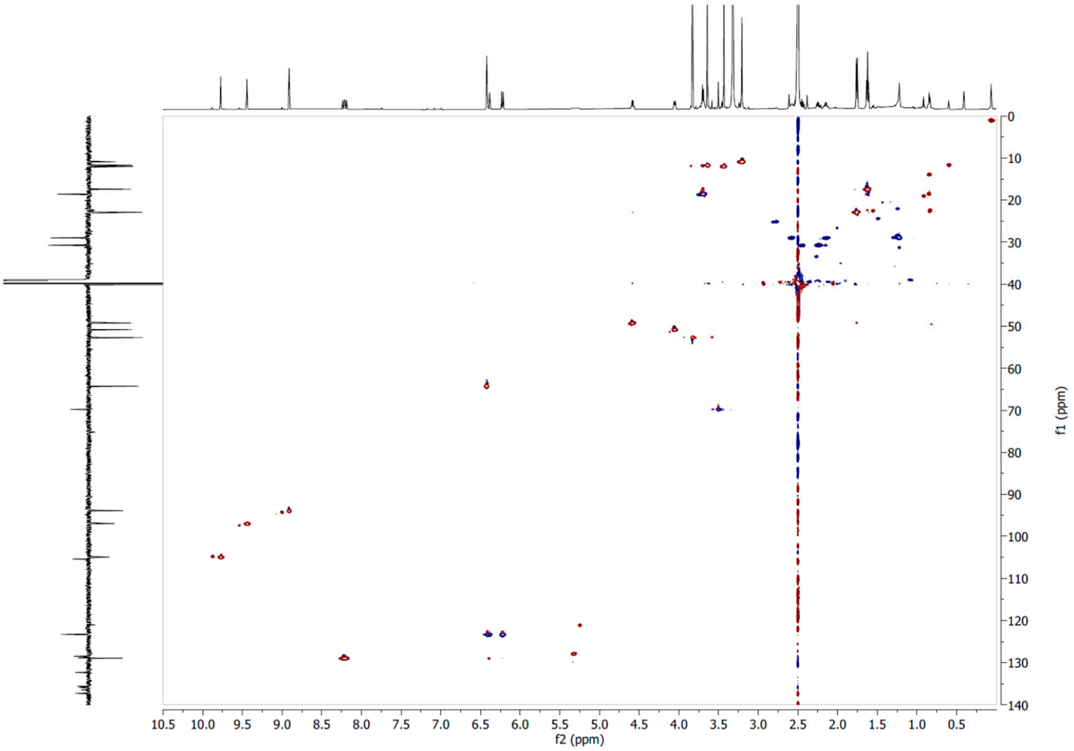

Supplementary Figure S15. Edited HSQC NMR spectrum of compound **3** in DMSO-*d*$_6$.

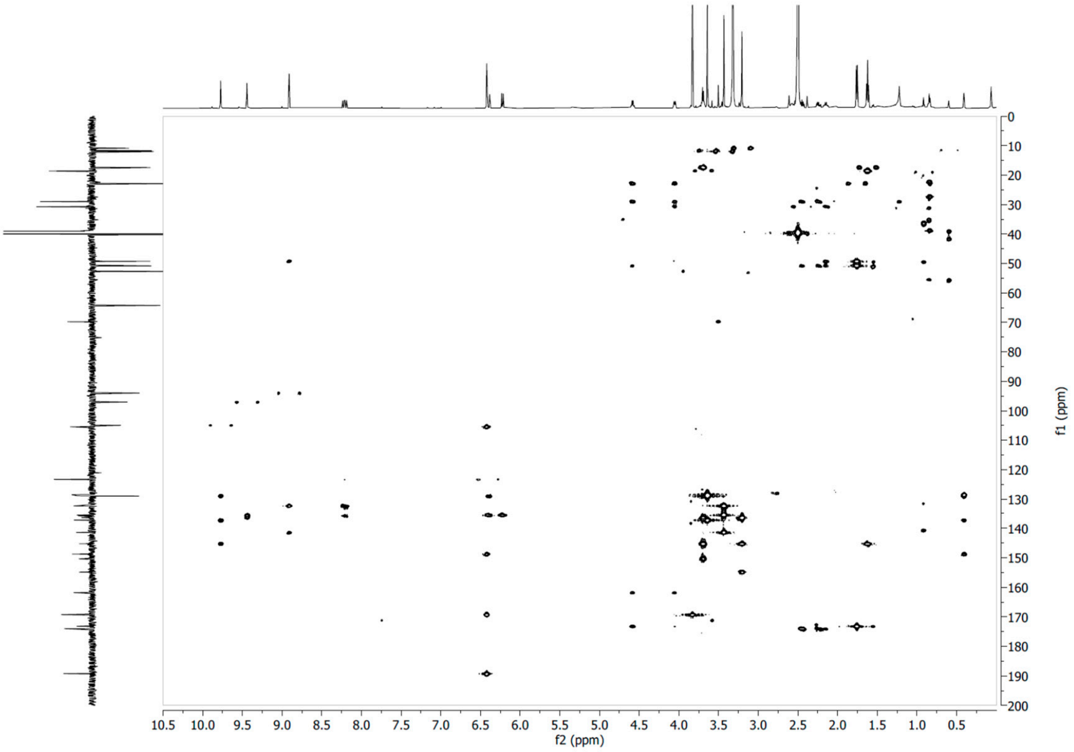

Supplementary Figure S16. HMBC NMR spectrum of compound **3** in DMSO-*d*$_6$.

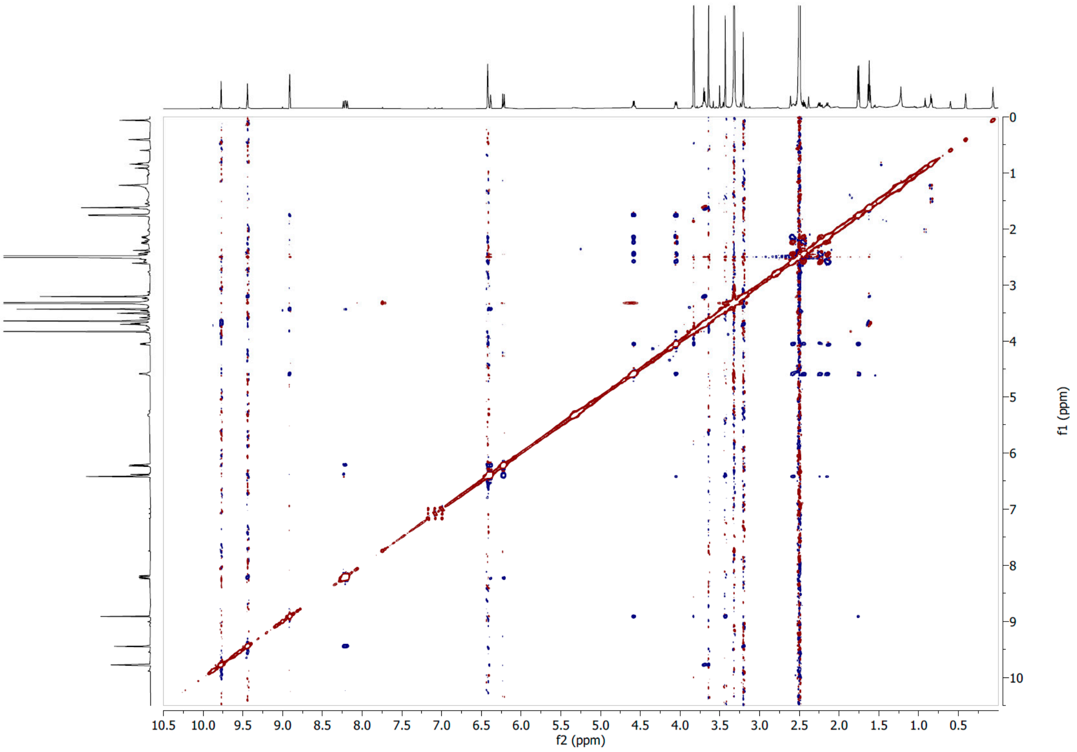

Supplementary Figure S17. ROESY NMR spectrum of compound **3** in DMSO-d_6.

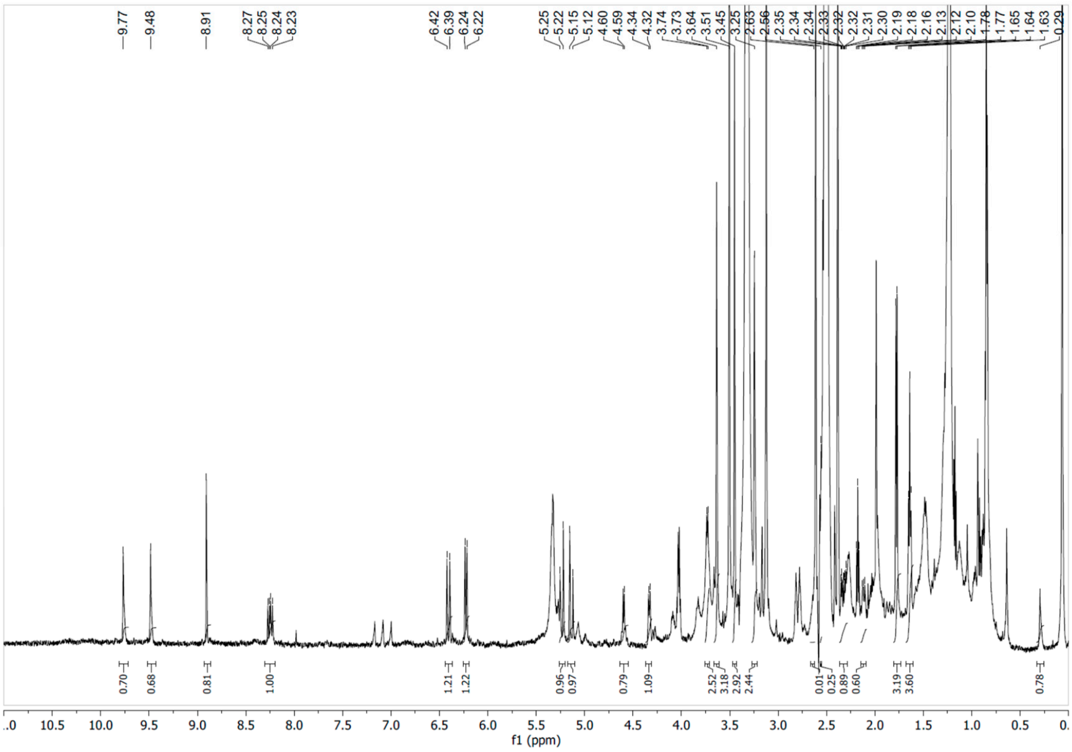

Supplementary Figure S18. ^{1}H NMR spectrum of compound **4** in DMSO-d_6 at 600 MHz.

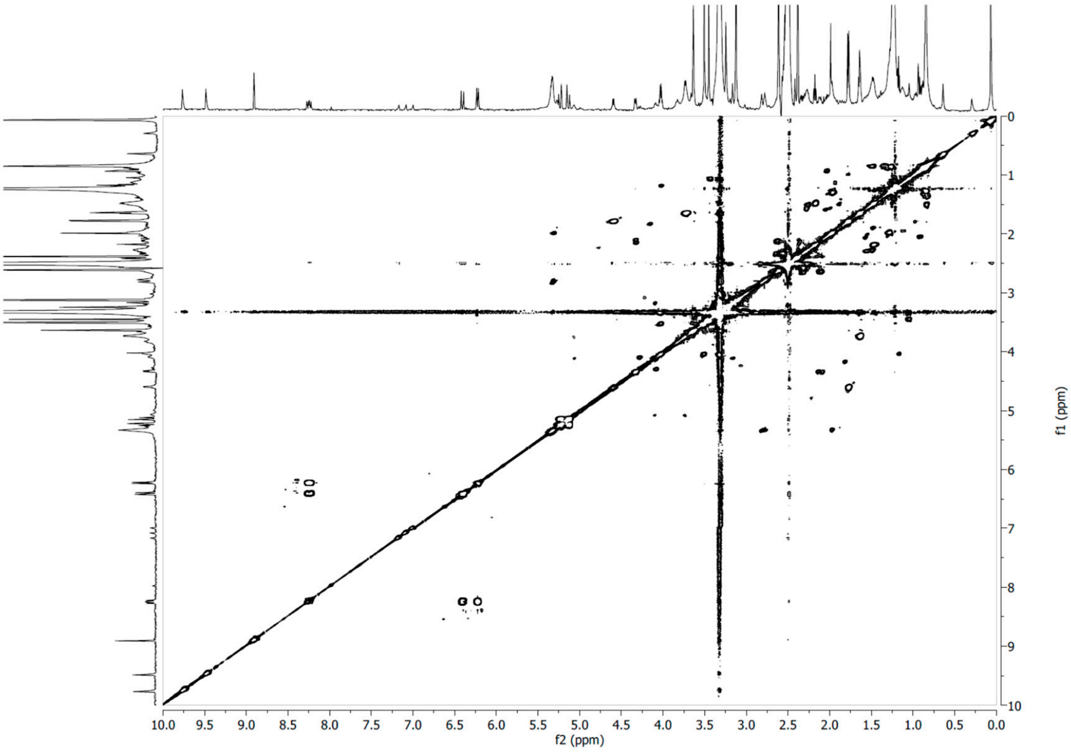

Supplementary Figure S19. COSY NMR spectrum of compound **4** in DMSO-d_6.

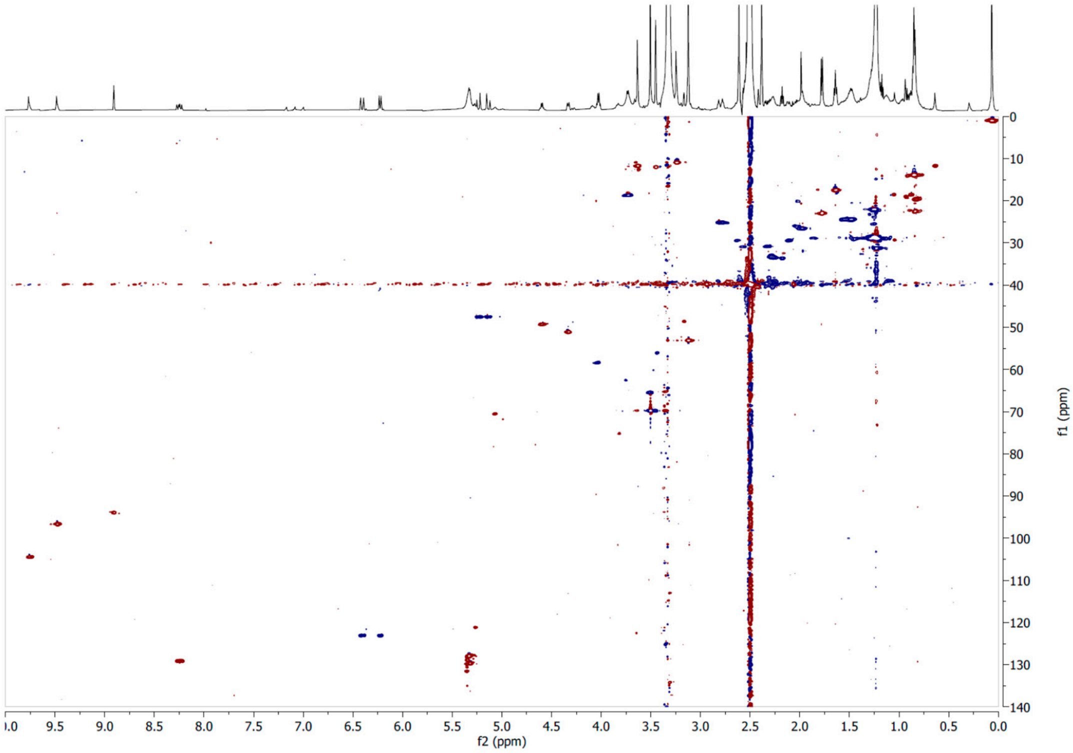

Supplementary Figure S20. Edited HSQC NMR spectrum of compound **4** in DMSO-d_6.

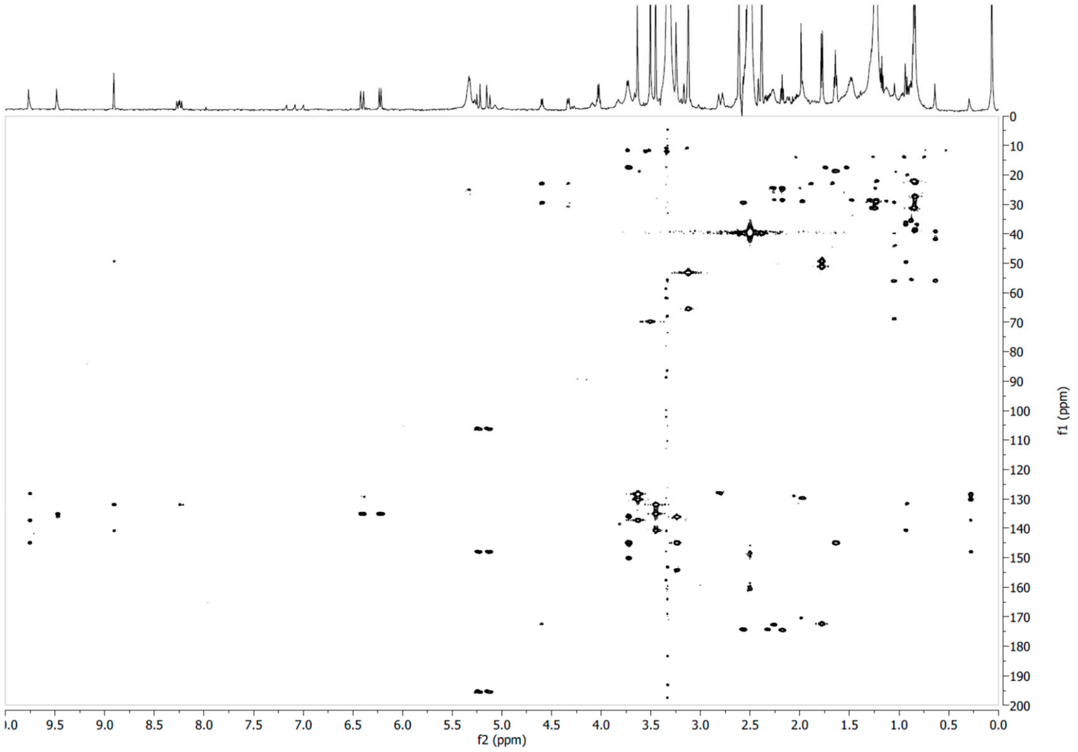

Supplementary Figure S21. HMBC NMR spectrum of compound **4** in DMSO-d_6.

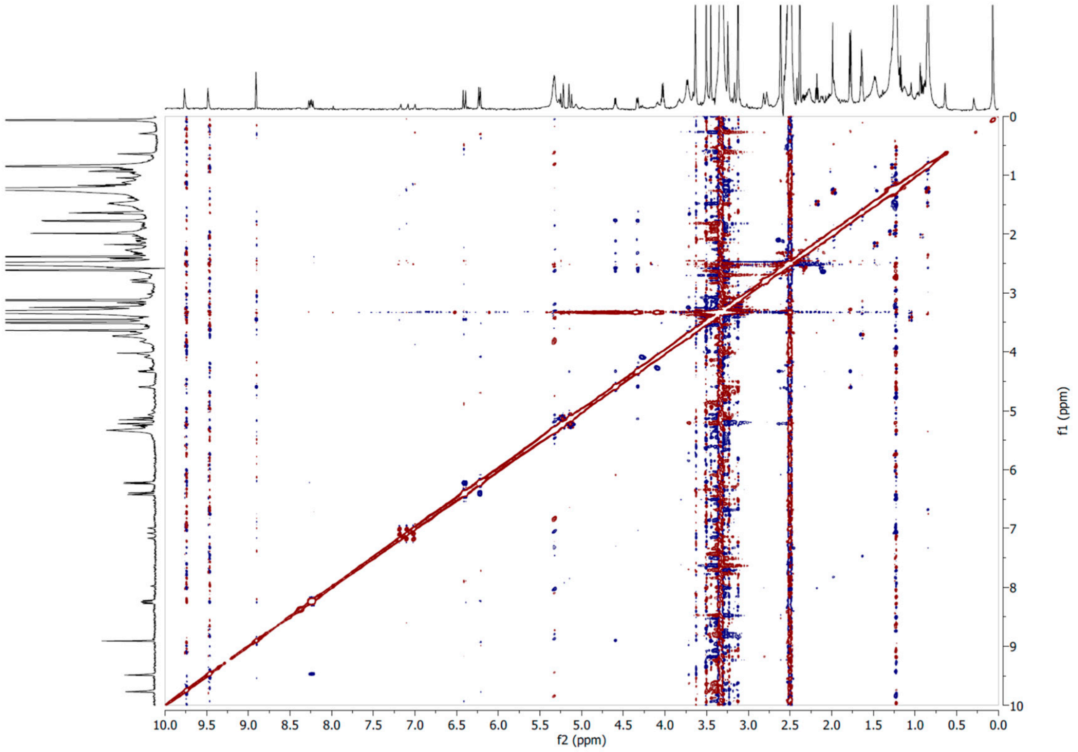

Supplementary Figure S22. ROESY NMR spectrum of compound **4** in DMSO-d_6.

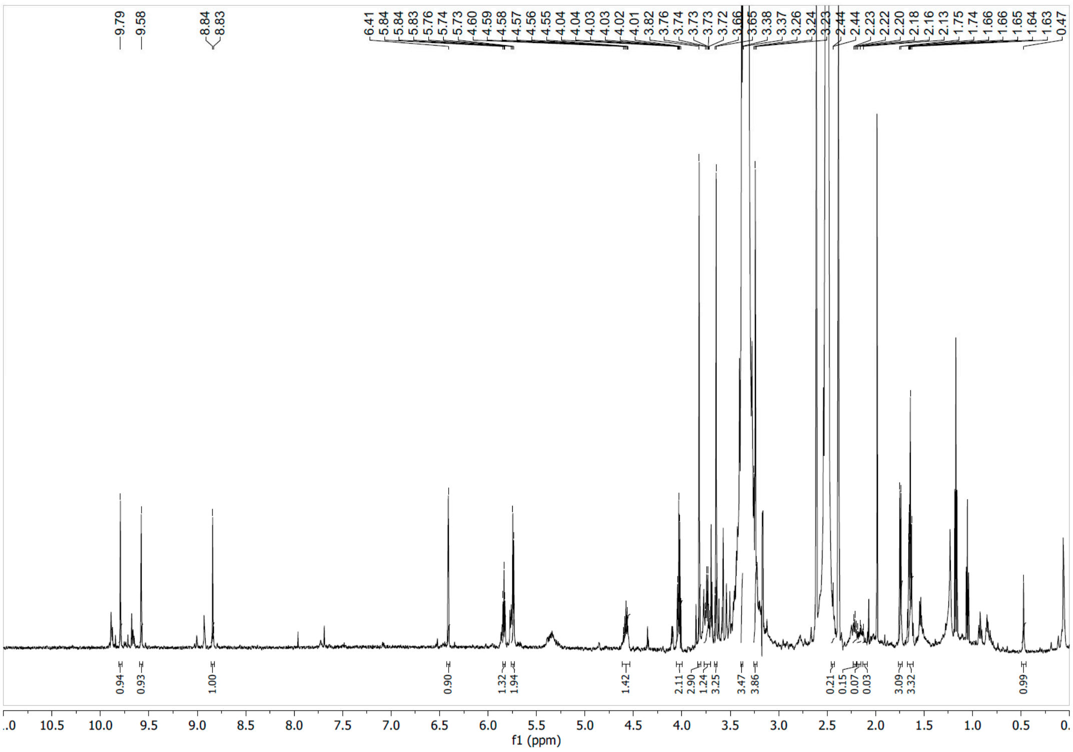

Supplementary Figure S23. ^{1}H NMR spectrum of compound **5** in DMSO-d_6 at 600 MHz.

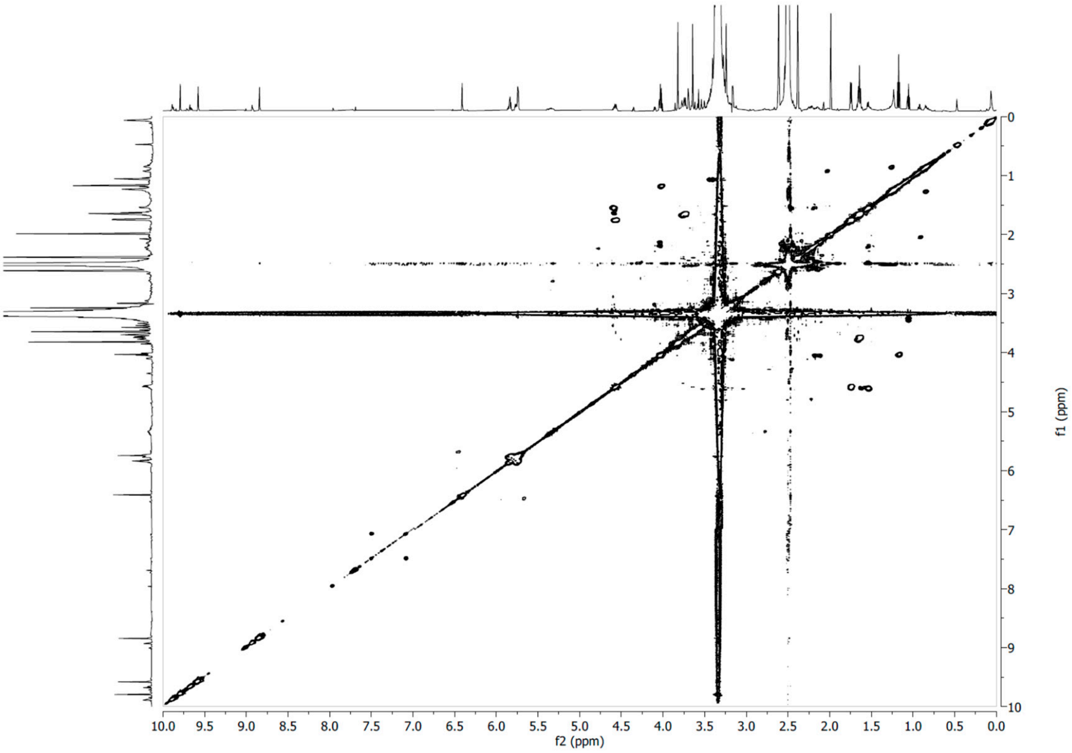

Supplementary Figure S24. COSY NMR spectrum of compound **5** in DMSO-d_6.

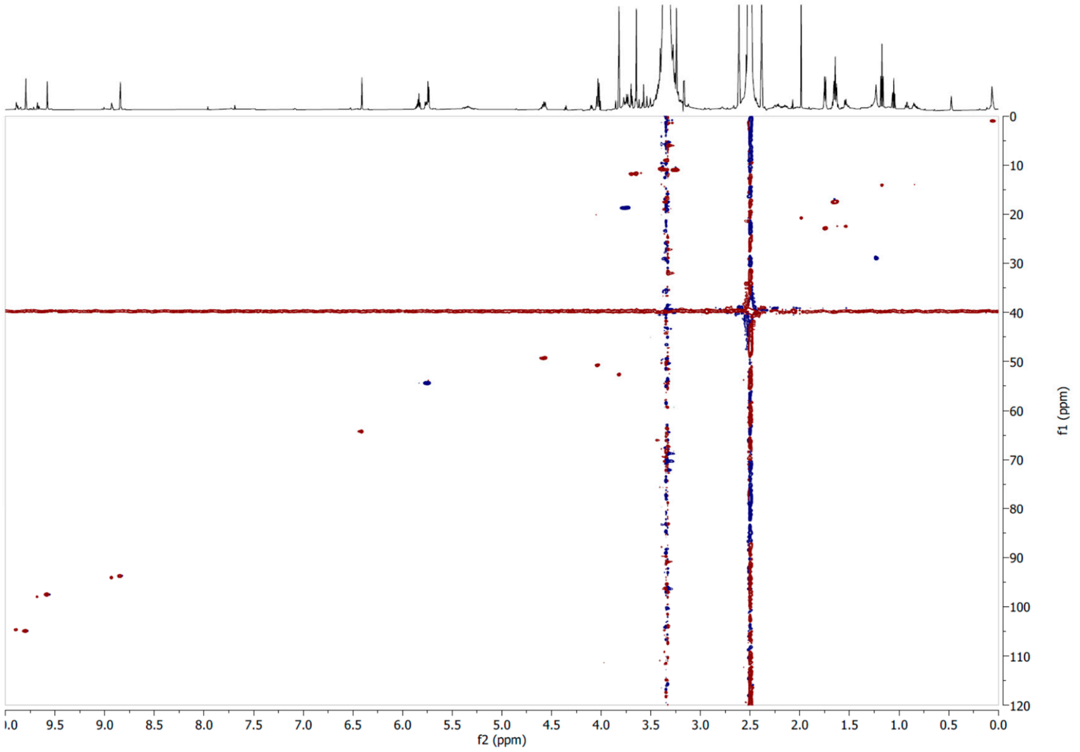

Supplementary Figure S25. Edited HSQC NMR spectrum of compound **5** in DMSO-d_6.

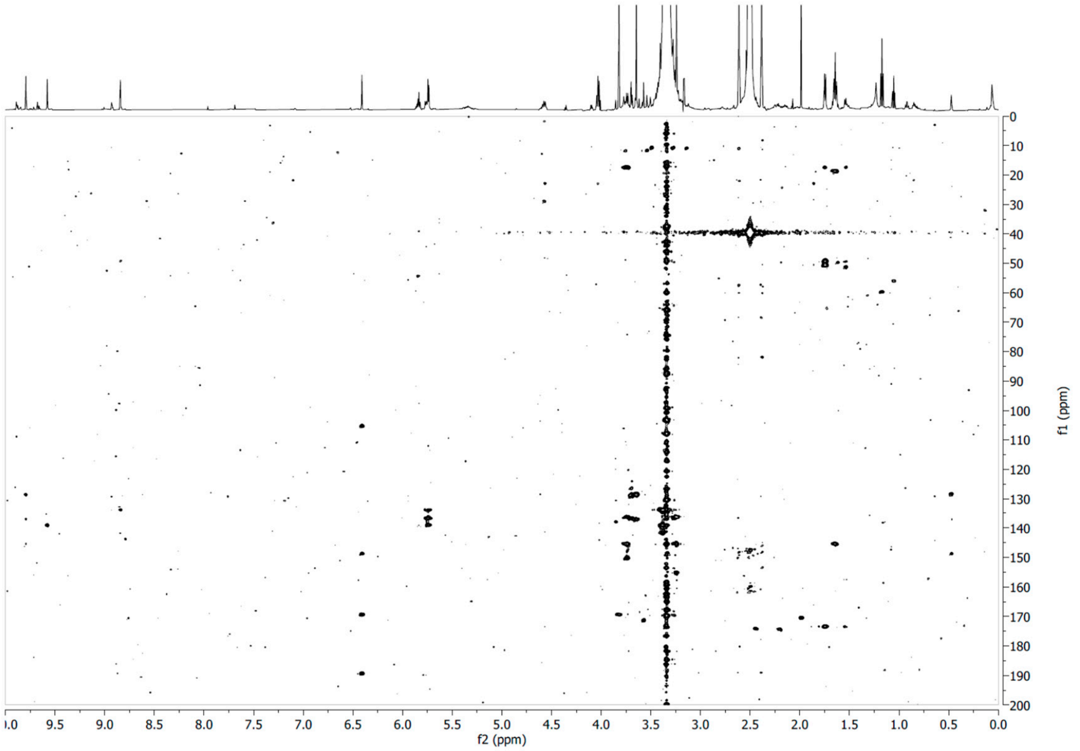

Supplementary Figure S26. HMBC NMR spectrum of compound **5** in DMSO-d_6.

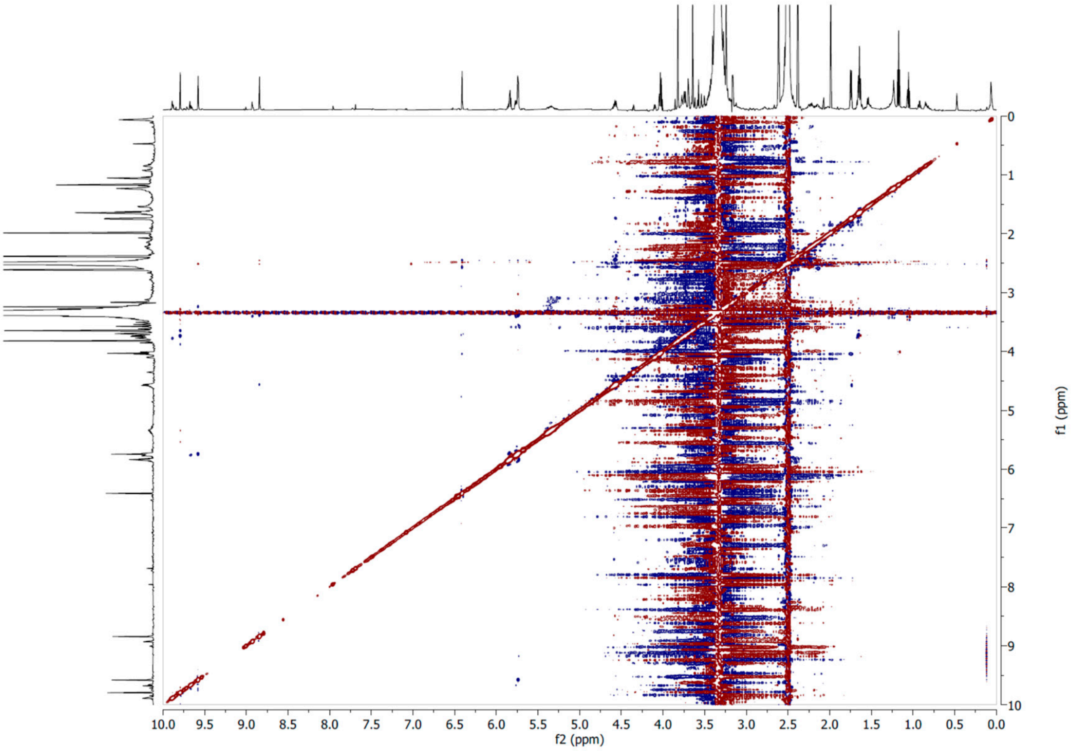

Supplementary Figure S27. ROESY NMR spectrum of compound **5** in DMSO-d_6.

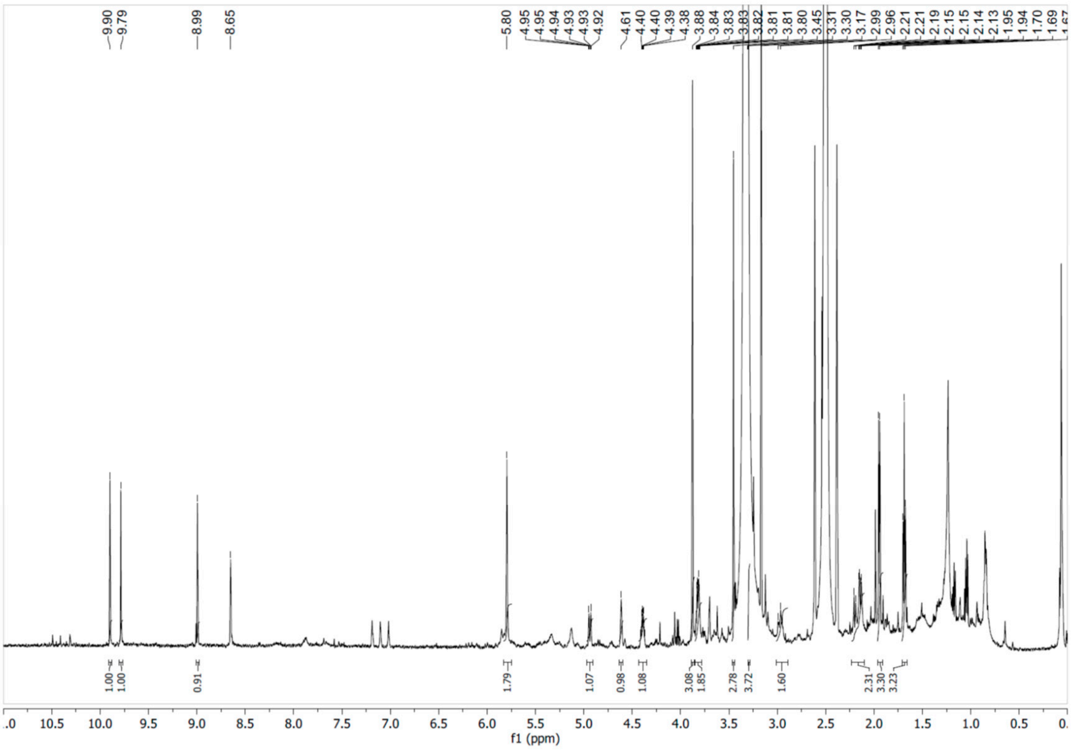

Supplementary Figure S28. ^{1}H NMR spectrum of compound **6** in DMSO-d_6 at 600 MHz.

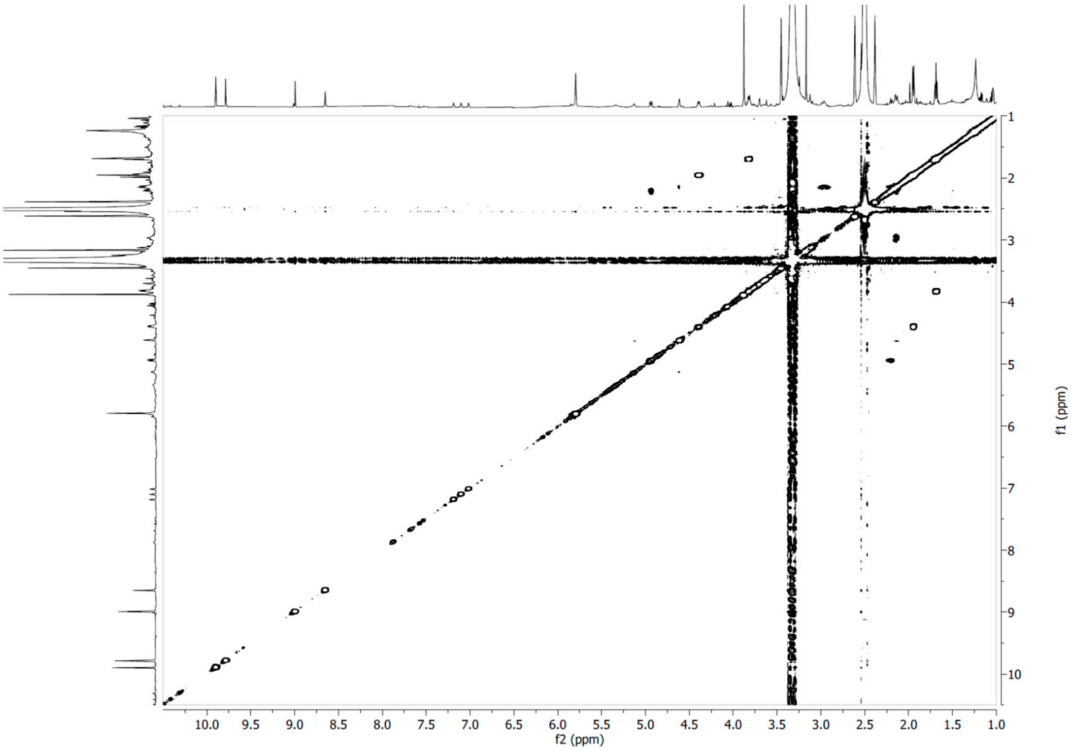

Supplementary Figure S29. COSY NMR spectrum of compound **6** in DMSO-d_6.

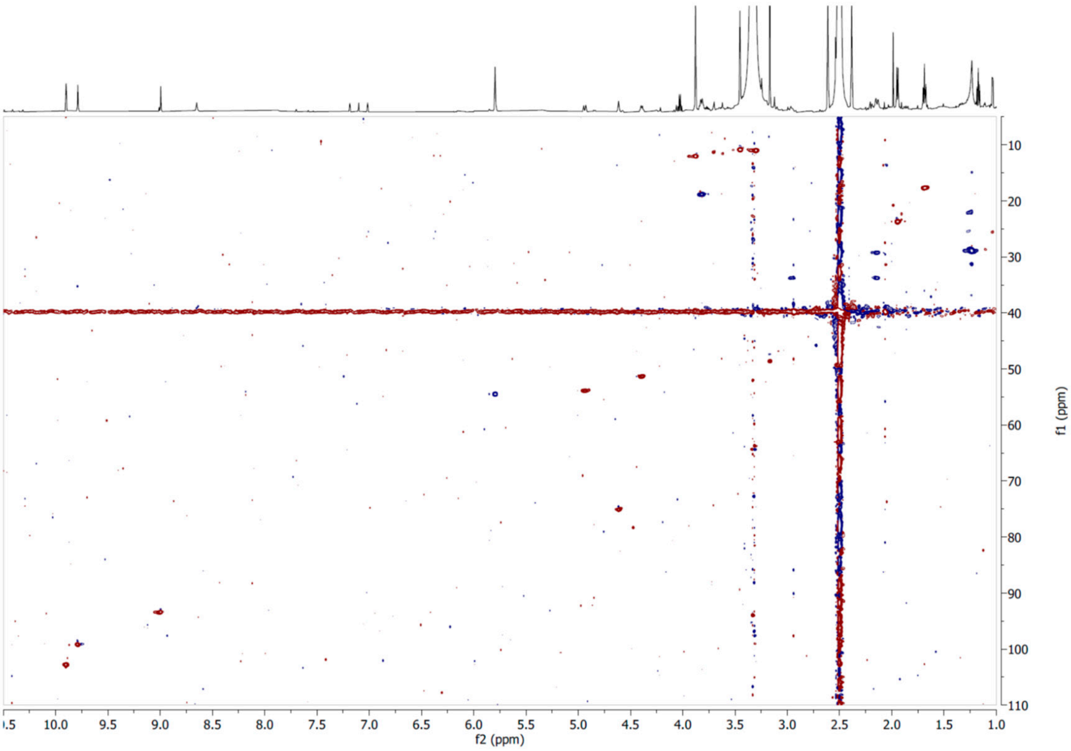

Supplementary Figure S30. Edited HSQC NMR spectrum of compound **6** in DMSO-d_6.

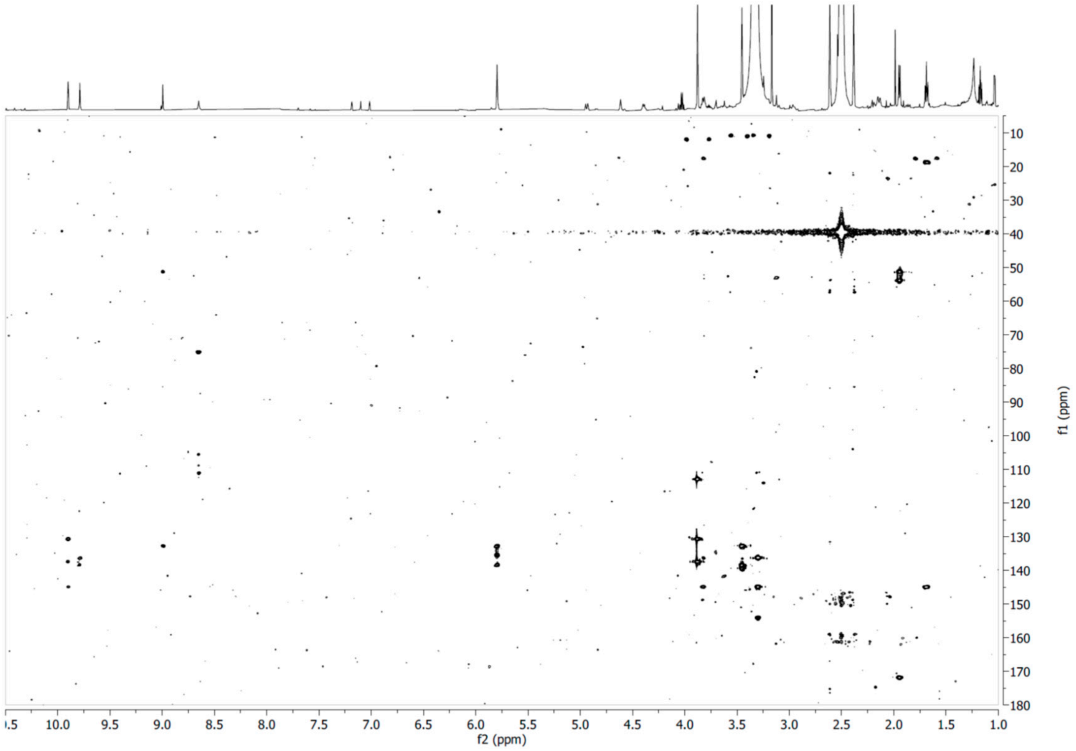

Supplementary Figure S31. HMBC NMR spectrum of compound **6** in DMSO-d_6.

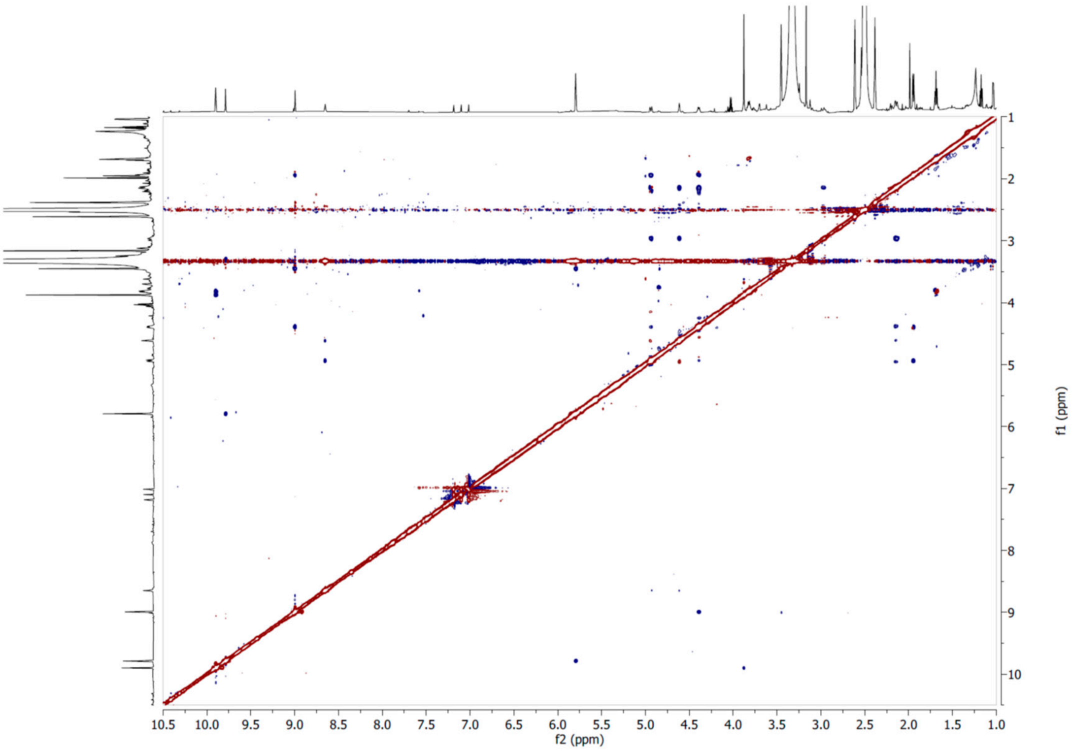

Supplementary Figure S32. ROESY NMR spectrum of compound **6** in DMSO-d_6.

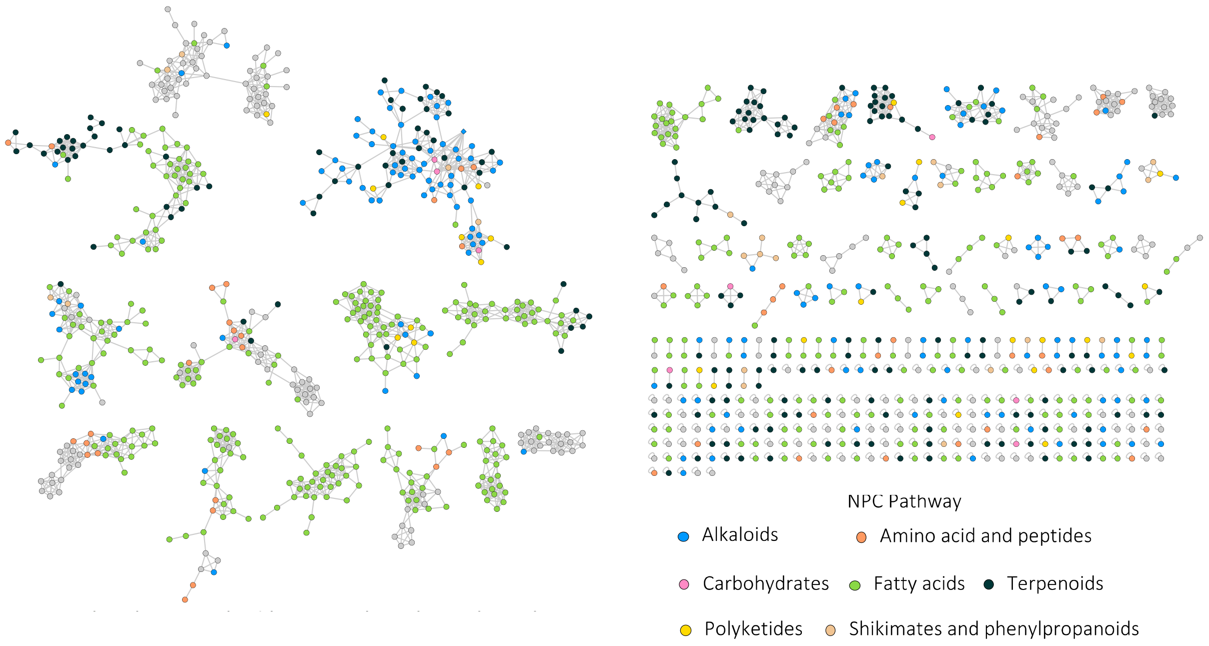

Supplementary Figure S33. Feature-based Molecular Network of the EtOAc and MeOH (butanolic fraction) extracts of *Ophiura sarsii*. Nodes are colored according to their predicted natural product pathway (NPClassifier).

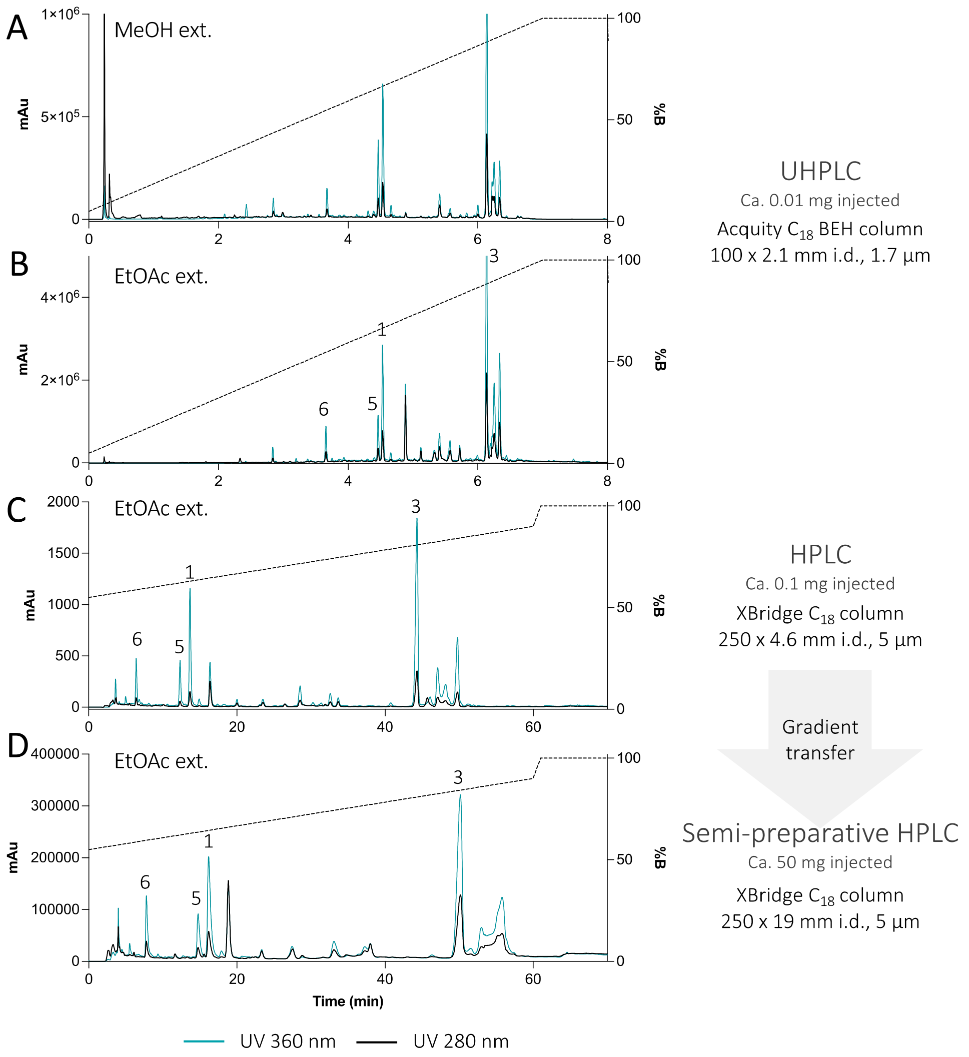

Supplementary Figure S34. (A, B) UHPLC-PDA-ELSD analysis of the raw MeOH and EtOAc extract of *Ophiura sarsii*. (C) Optimized HPLC-PDA-ELSD analysis of the EtOAc extract of *O. sarsii*. (D) semi-preparative HPLC-UV analysis after gradient transfer using a dry load injection.

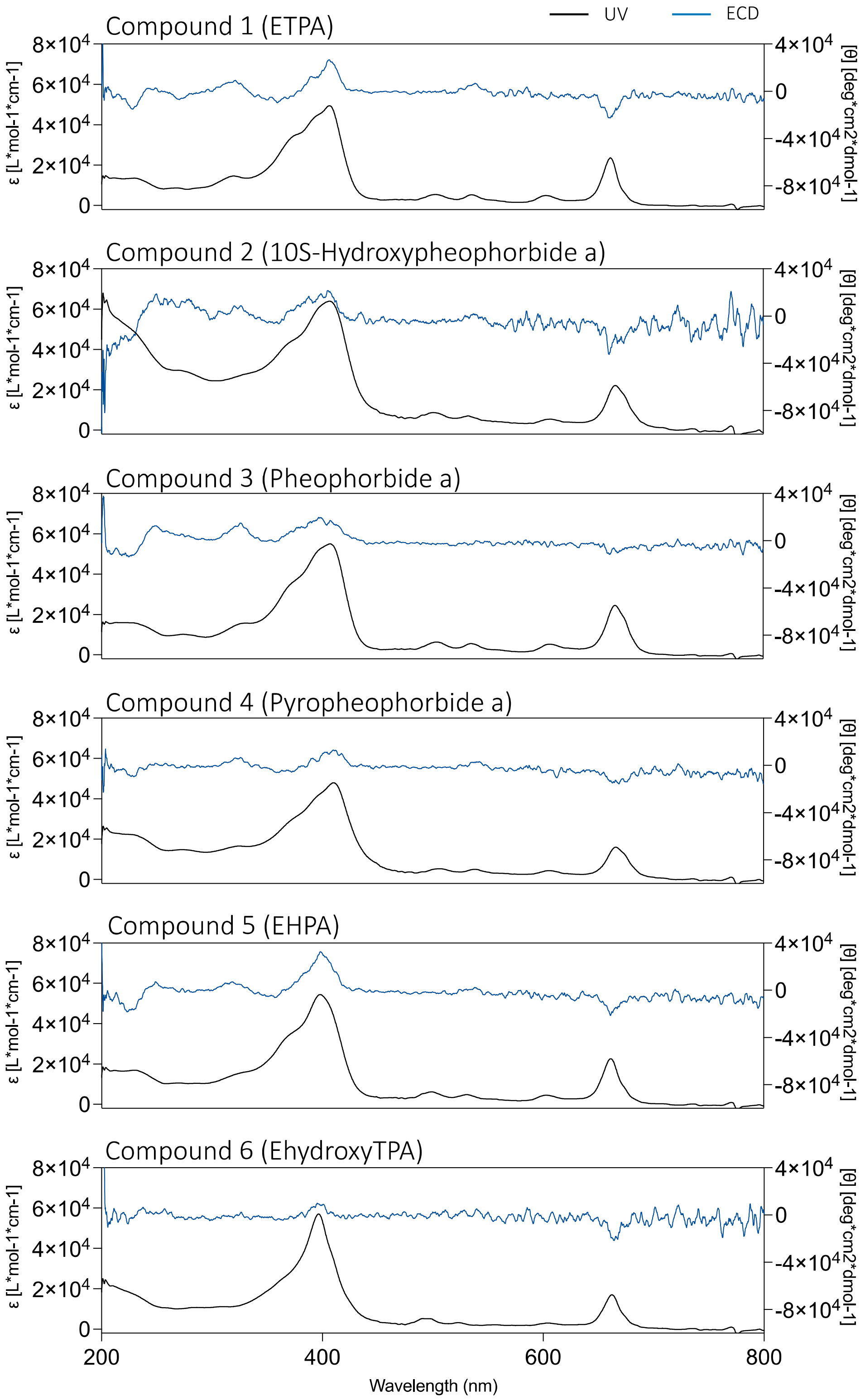

Supplementary Figure S35. UV-Vis and ECD spectra of the isolated compounds.

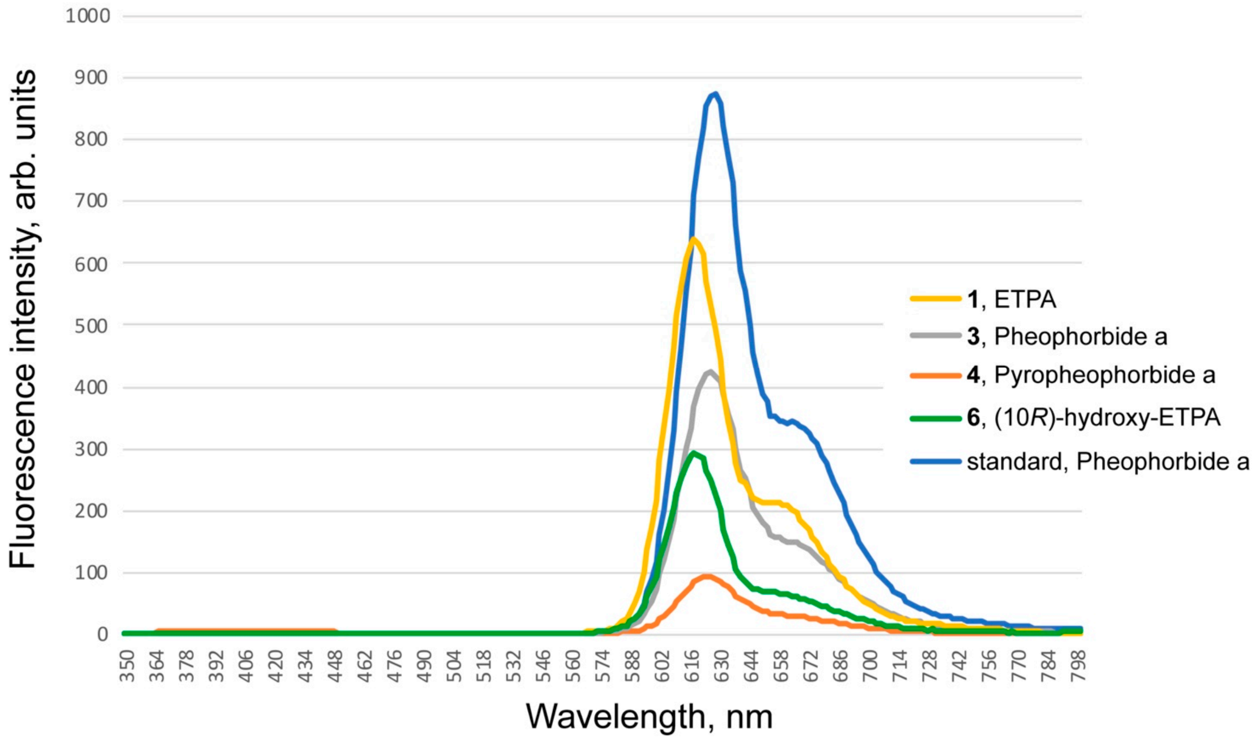

Supplementary Figure S36. Fluorescence spectra of compounds **1** (ETPA), **3** (Pheophorbide a), **4** (Pyropheophorbide a), and **6** ((10*R*)-hydroxy-ETPA), along with that of commercial standard (Pheophorbide a) after excitation at 350 nm.

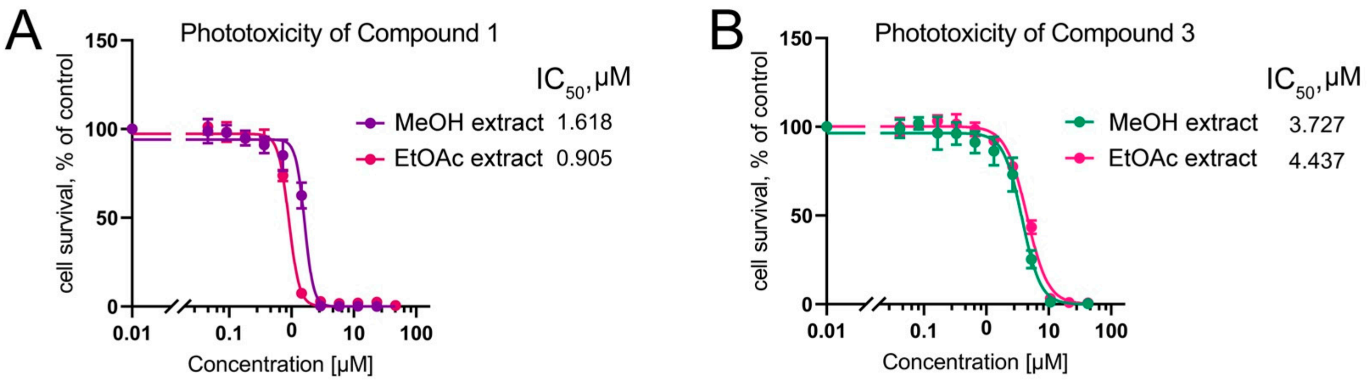

Supplementary Figure S37. Phototoxicity of compounds **1** (ETPA, A) and **3** (Pheophorbide a, B) isolated from MeOH and EtOAc extracts.
